# Missing values and inconclusive results in diagnostic studies – A scoping review of methods

**DOI:** 10.1177/09622802231192954

**Published:** 2023-08-09

**Authors:** Katharina Stahlmann, Johannes B Reitsma, Antonia Zapf

**Affiliations:** 1 Institute of Medical Biometry and Epidemiology, 37734 University Medical Center Hamburg-Eppendorf, Germany; 2 Julius Center for Health Sciences and Primary Care, University Medical Center Utrecht, Utrecht University, the Netherlands

**Keywords:** Diagnostic study, accuracy, missing values, inconclusive, reference standard, index test, sensitivity and specificity

## Abstract

Most diagnostic studies exclude missing values and inconclusive results from the analysis or apply simple methods resulting in biased accuracy estimates. This may be due to the lack of availability or awareness of appropriate methods. This scoping review aimed to provide an overview of strategies to handle missing values and inconclusive results in the reference standard or index test in diagnostic accuracy studies. Conducting a systematic literature search in MEDLINE, Cochrane Library, and Web of Science, we could identify many articles proposing methods for addressing missing values in the reference standard. There are also several articles describing methods regarding missing values or inconclusive results in the index test. The latter encompass imputation, frequentist and Bayesian likelihood, model-based, and latent class methods. While methods for missing values in the reference standard are regularly applied in practice, this is not true for methods addressing missing values and inconclusive results in the index test. Our comprehensive overview and description of available methods may raise further awareness of these methods and will enhance their application. Future research is needed to compare the performance of these methods under different conditions to give valid and robust recommendations for their usage in various diagnostic accuracy research scenarios.

## 1 Introduction

Accurate diagnostic tests are a crucial prerequisite to diagnosing patients correctly and assigning them to the right treatment.^1^ A false diagnosis can have severe consequences for the patient's psychological well-being and therapeutic decisions.^1^ Diagnostic tests are evaluated in so-called diagnostic accuracy studies which compare a reference standard—defined as “the best available method for establishing the presence or absence of the target condition”^2^—with a newly developed test, the index test. Sometimes, the accuracy of two or more index tests is compared in the same study; this is then referred to as a comparative diagnostic accuracy study.^3^ If a reference standard classifies individuals error-free into those with and without the target condition, some researchers also call it a gold standard.^4^ To avoid confusion, we will stick to the term reference standard throughout the article.

The accuracy of an index test with a binary result can be described by its sensitivity (the probability of a positive test result given the person has the target condition) and its specificity (the probability of a negative result given the person does not have the target condition). For continuous tests, the receiver operating characteristic (ROC) curve and the area under the ROC curve (AUC) can be estimated.^5^ The AUC describes the “probability that, when presented with a randomly chosen patient with the disease and a randomly chosen patient without disease, the results of the diagnostic test will rank the patient with disease as having higher suspicion for disease than the patient without disease.”^6^

Hitherto, most research has been focused on methodological issues of missing values in intervention studies, not diagnostic accuracy studies. Despite all efforts to prevent missing values in the design and conduct of a study,^7^ missing values are likely to occur and can pose a threat to the validity of the results.^2^ In addition to being missing, test results can be inconclusive, if, for instance, the result is not interpretable or neither positive nor negative.^8^ If not appropriately considered, missing values and inconclusive test results can lead to biased estimates.^9^ Thus, it is essential to address them properly in the statistical analysis.

To date, most research has proposed methods for missing values in the reference standard or for addressing an imperfect reference standard (a reference standard that will make errors when classifying participants as having the target condition or not, so the true status is still “uncertain or unknown”). Nonetheless, missing values and inconclusive results are often excluded from the analysis of diagnostic studies^10^—for example, in 40% of studies evaluating CT of coronary angiography^11^—or analyzed by using simple imputation methods, for example, positive, negative imputation, or intention-to-diagnose.^11^ Moreover, Shinkins et al.^10^ noted that only a third of diagnostic studies included in systematic reviews between 2005 and 2011 had reported the occurrence and handling of inconclusive results adequately. Similarly, only a third of diagnostic studies in the field of acute care medicine reported the handling of missing values and inconclusive results.^12^ This is in contrast with the STARD guideline which explicitly states that missing values and inconclusive results should be displayed transparently and recommends a clear description of the handling of such results.^2^ Reasons for ignoring or applying simple methods for missing values and inconclusive results in the analysis may be uncertainty or lack of knowledge among applied researchers about which approach to use in which scenario. One explanation for this may be that there are only a few articles that have summarized and compared the existing approaches.

Therefore, this scoping review aims at giving an overview of the existing approaches to handle missing values and inconclusive test results in both the reference standard and the index test in diagnostic accuracy studies. Furthermore, these approaches are evaluated and research gaps are identified. This paper is structured as follows: In Section 2, we describe the definition and reasons for the occurrence of missing values and inconclusive results in diagnostic studies and give an overview of possible related biases. Subsequently, we present the methodology of our review in Section 3. The results of our review are summarized in Section 4 and discussed in Section 5. Finally, our key points are summarized in a short conclusion.

## 2 Missing values and inconclusive results in diagnostic accuracy studies

Common approaches to handling missing values in intervention studies cannot simply be used in diagnostic studies as these feature some unique design aspects. For example, while intervention studies examine relative effect measures for outcomes that are relatively easy to determine (such as death, hospital admission, or laboratory measures), diagnostic accuracy studies estimate absolute probabilities and determine the presence or absence of a target condition (which is technically and ethically more complex). In addition, diagnostic studies sometimes feature nested designs and clustered data which pose a challenge for the analysis, especially when considering missing values and inconclusive results.^13^ For instance, this may emerge if multiple raters examine all test results or if multiple lesions within a patient can be present and evaluated. Clustered data are a central issue in the paired design/within-subject design, in which all participants receive two (or more) index tests as well as the reference standard. In contrast, in the parallel group design, study participants are randomized into two groups one of which receives only one index test and the other group only the other test, with the reference standard being applied in both groups. However, the paired design is preferred as it allows a within-person comparison that can reduce variability. Nevertheless, it also bears the specific problem of conditional dependence of the index tests, or an index test and an imperfect reference test. Many methods assume conditional independence—that is, errors of both tests are not correlated or tests are independent conditional on true presence/absence of target condition—which might be true under certain circumstances but not always.^14^

Generally, missing values can be missing completely at random (MCAR), at random (MAR), or not at random (MNAR).^15^ According to this classification, Begg et al.^16^ defined in case of uninterpretable results that these may be uninterpretable at random, dependent on the target condition alone and dependent on the target condition and correlated with the underlying test result. To assess which of these three patterns is likely, to choose an adequate analysis strategy, and thereby, avoid possible biases, it is helpful to know the reasons for missingness or inconclusiveness. Reasons for missingness can be manifold and may lie in the study planning, conduct, or participant characteristics. Therefore, the present missingness mechanism cannot be deduced from the data itself. A causal diagram can improve the understanding of the association between (measured or unmeasured) variables and missingness^17^ and an extensive data examination can help to gain insights into the missingness patterns and possible mechanisms.^17,18^ For instance, it may be useful to examine the association between measured variables and missingness (yes vs. no) by means of logistic regressions, or to explore missingness patterns graphically. Nonetheless, the analysis will still be based on assumptions of missingness mechanisms that cannot be fully verified. While conducting a complete case analysis can often result in highly biased results and loss of efficiency,^19^ applying an inadequate strategy for dealing with such results will also be likely to produce bias. As an example, Ma et al.^20^ showed that using the intention-to-diagnose approach or positive/negative imputation leads to substantially biased estimates for accuracy. In the following, we will shortly describe various scenarios in which missing values and inconclusive results can occur as well as possible biases which may be related to these scenarios.

### 2.1 Missing values and related biases

As per the definition, a missing value simply means that the test result is not present. Missing values can arise in the reference standard, in the index test, or in covariates and may be due to the unavailability of study equipment, individuals’ refusal to undertake a testing procedure, or the decision to verify only a selection of individuals owing to an invasive reference standard method,^7,21^ The latter can lead to partial or differential verification bias.^5,19^ The partial verification bias—also known as a referral, ascertainment, or work-up bias—describes the case that not all individuals receive the reference standard and those with a positive index test are more likely to be verified than those with a negative result. Excluding subjects with missing verification from the calculation of accuracy parameters leads to an overestimation of sensitivity and an underestimation of specificity.^9,19^ Differential verification can emerge if the unverified individuals receive a different reference standard and both reference standards give different results for the true target condition status (present/not present).^9,19^ In this case, the direction of bias can vary.^9^

The application of an imperfect reference standard can generally lead to bias. Its direction and magnitude depend on the conditional (in)dependence of the index test and reference test^9,21^ although Whiting et al.^9^ observed in empirical studies that sensitivity is often stronger affected than specificity. If, for example, an imperfect reference standard is defined as an expert panel, whose members take all information into account to define the presence or the absence of the target condition, its members must be blinded toward the result of the index test to avoid incorporation bias.^21^ Otherwise, incorporation bias may lead to overestimated estimates of accuracy.^5,21^

### 2.2 Inconclusive results and related biases

Inconclusive results can be distinguished into uninterpretable (e.g. because of poor image quality), and intermediate (the result lies between clearly positive or negative values).^8,10,16^ Within intermediate results, indeterminate results represent a subset and are defined as results that have a likelihood ratio of 1 and do not alter the probability of the target condition.^8,10,16^ Shinkins et al.^10^ considered uninterpretable results invalid (they cannot be interpreted regarding the probability of the target condition) and, thus, similar to (but not the same as) missing values. In contrast, they deem indeterminate and intermediate results valid as they give information about the index test value even though they cannot clearly indicate whether an individual subject is positive or negative. Although uninterpretable and missing test results bear some similarities to the analysis, they have to be distinguished.^10^ Simply speaking, “missing” refers to a case where no test result has been recorded, while “uninterpretable” defines a case where the test was performed, but the result is invalid (it cannot be interpreted regarding the probability of the target condition).^10^ Consequently, Shinkins et al.^10^ proposed to handle both types of inconclusive results differently in the statistical analysis.

Another important aspect in deciding on an appropriate method is the reason for inconclusiveness, in particular, whether it is related to the target condition. For instance, uninterpretable test results may be a result of false conduct of the test or due to a clinical characteristic of the participant which affects the test. Hence, reasons for lacking interpretability are rather associated with the objective quality of a test (i.e. its intrinsic properties).^10^ If the reason for lacking interpretability is clearly unrelated to the probability of the target condition, the test may be repeated in most cases. However, the reason can sometimes be related to the probability of the target condition and is, therefore informative.

Indeterminate and intermediate results, in particular, can be related to a spectrum bias.^19,22^ According to the EMA,^1^ confirmatory trials must assess the index test in the intended-to-use/target population to make valid inferences about accuracy in clinical practice. This population consists of those individuals who should receive the index test in clinical practice.^1,5^ Omitting all participants with indeterminate or intermediate results (i.e. individuals in whom the target condition may not have manifested fully or who may be in transition from healthy to diseased) from the analysis can result in an analysis population that is not representative of the target population anymore. That is to say, only those subjects with clear presence or absence of the target condition are used for the analysis, and, consequently, accuracy may be overestimated.^5,19^

Therefore, it is crucial to explore reasons for and patterns of missingness and inconclusiveness and to address missing values and inconclusive results adequately in the statistical analysis to minimize the risk of biased results.

## 3 Methods

Our scoping review aimed at providing an overview of existing methods to handle missing values and inconclusive results in the reference standard and/or index test in the statistical analysis of diagnostic accuracy studies. Furthermore, we sought to evaluate the current state, compose a selection of diagnostic studies which have applied the proposed strategies, and identify research gaps in this area. In writing this article, we followed the PRISMA-ScR guideline extension for scoping reviews. The checklist can be found in the Supplemental Material.

First, the focus was on identifying methodological articles that developed, described, or compared strategies to handle missing values or inconclusive results in diagnostic studies. Original diagnostic research was also included if it discussed the performance of a specific method (in detail). For this purpose, a systematic search was conducted (described below). In the next step, original diagnostic research that applied one or more of the proposed strategies was identified by additional citation searching via Google Scholar.

### 3.1 Search strategy and selection of articles

To compile all relevant literature on strategies to handle missing values or inconclusive results, one author (Katharina Stahlmann) conducted a systematic search in MEDLINE, Cochrane Library, and Web of Science in April 2022 (without further time restriction). The same search strategy was adapted to the respective database (see Supplemental Material). All articles were imported to Endnote 20.4 for subsequent screening. After removing duplicates, Katharina Stahlmann screened first the titles/abstracts of all identified articles and then the full texts of publications considered eligible on a title/abstract basis. Full texts of 12 articles could not be retrieved for the full-text screening. In addition, citation searching was performed to identify other eligible articles from the reference lists of the included articles or articles citing the included ones via Google Scholar. Uncertainties regarding inclusion or exclusion were discussed with a second researcher (Antonia Zapf). The following inclusion and exclusion criteria were applied in the screening process.

### 3.2 Eligibility criteria

Articles were included if they

- discussed strategies to deal with missing values in either the index test or reference standard, inconclusive (indeterminate, intermediate, uninterpretable, non-evaluable) test results, or an imperfect reference standard. This included also articles on differential and partial verification bias,
- focused on diagnostic accuracy studies,
- assessed common outcome parameters of diagnostic accuracy studies, such as ROC/AUC, sensitivity, specificity (or were a review of different strategies without focusing on specific outcome parameters) and
- were published in English or German.

Articles were excluded if they

- did not describe the analysis of diagnostic studies,
- mentioned a method to analyze missing values but did not discuss its performance,
- were a diagnostic benefit study (diagnostic studies which examine the down-stream consequences of test results, for example, the impact on physicians’ decisions, patient outcomes, or costs).

### 3.3 Data synthesis

Data from the included articles were extracted by one researcher (Katharina Stahlmann) into a structured Excel data sheet and then discussed with a second researcher (Antonia Zapf). The extraction sheet included the items: author(s), year, type of study (original research, review, and methodological paper), outcome assessed, number of index tests, examination of missing values, or inconclusive results, missing values/inconclusive results in the reference standard, or an imperfect reference standard (yes and no), missing values/inconclusive results in the index test (yes and no), description of the proposed method, missingness patterns (MCAR, MAR, and MNAR), advantages, disadvantages, and additional notes. Then, the data were synthesized narratively.

## 4 Results

**Figure 1.**
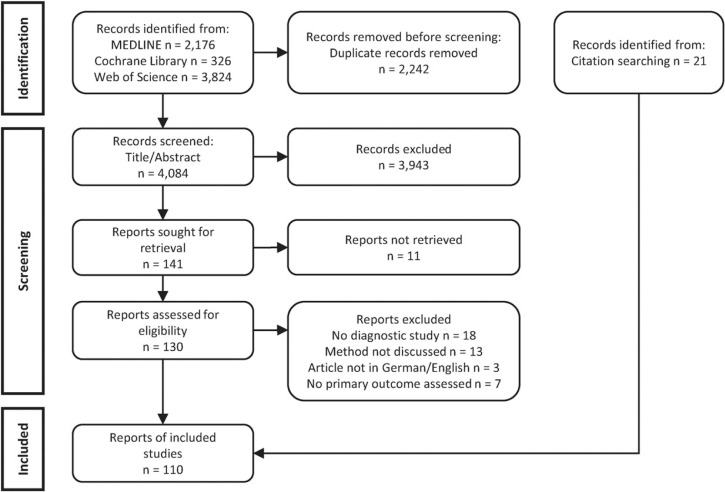
Flow diagram of the search and selection process.

In summary, 4086 articles were found via the systematic database search of which 98 were included in the review after the screening process. In addition, 21 reports were identified through citation searching amounting to a final number of 110 included articles (Figure 1). Bibliographic information of the articles excluded in the full-text screening and reasons for exclusion are given in the Supplemental Material. As this scoping review aimed at providing an overview of methods to handle missing values in diagnostic studies, we classified the identified methods and described them in general terms in the following. More details on each method, including strengths, weaknesses, and tools for their implementation, can be found in the Supplemental Material. A statistical explanation of each method is beyond the scope of this review and can be found in the original publications.

### 4.1 Missing values in the reference standard

A substantial number of articles (*n*  =  67) dealing with missing values in the reference test or with an imperfect reference test could be identified. The previous reviews^4,23,24^ provided a comprehensive summary of methods proposed in this context. Therefore, we will describe these methods shortly and refer to these reviews for further details. A detailed description of each method and its strengths and weaknesses can be found in the Supplemental Material of Chikere et al.^4^ Approaches to handling missing values in the index test and inconclusive results will be described in more depth in Sections 4.2to 4.4.

Missing values in the reference standard, also known as partial verification, can be addressed by adjustment or bias-correction methods or by imputation.^4,23,24^ When using these approaches, it is necessary to know the underlying missingness pattern to construct a correct imputation model and produce valid estimates.^23,24^ This may, for instance, be the case in an a priori planned partial verification design.^23^ In small samples or studies with a high proportion of missing reference standard results, imputation models, particularly multiple imputation (MI), were shown to be more robust than correction methods.^23^ Employing a second reference test for those individuals not verified with the first one—differential verification—is another possibility.^4,23,24^ However, it is likely to lead to differential verification bias whose direction depends on the proportion verified by the second test, test properties, the measurement error of the second test, and the mechanism of verification. Often, the probability of verification is dependent on the index test result and therefore, not completely at random.^23^ Consequently, this verification mechanism has to be considered in the statisticalanalysis.

Other methods exist for dealing with an imperfect reference standard. If the accuracy parameters of this imperfect reference standard are known, the diagnostic parameters of the index test can be corrected.^4,23,24^ There are several different correction methods available most of which assume conditional independence of the reference standard and index test. If conditional dependence is present and ignored or if the accuracy of the reference test is estimated incorrectly, accuracy estimates of the index test can be biased.^23,24^ Another option is to construct a composite reference standard of two reference tests. A definition of the presence of the target condition (e.g. present if both reference tests are positive, if only one of both is positive, or other combinations) must be set a priori to enhance transparency and prevent bias. Furthermore, the composite reference standard should have better accuracy in classifying individuals with and without the target condition than each test alone.^23,24^ This approach can be quite efficient if only one of both tests must be positive to define the presence of the target condition and, consequently, redundant testing and related participant burden can be avoided.^23^ Nonetheless, it bears the risk of residual error and, moreover, there are often no universally agreed decision rules for the presence of the target condition under a composite reference standard. Moreover, using a composite reference standard is likely to result in biased estimates in many scenarios.^25^

The composite reference standard must not be confused with a discrepant analysis. In discrepant analysis, patients receive the index test followed by the first reference test, and only those with discordant results between the first reference test and index test receive will receive the second reference test, which is typically the more expensive or invasive reference test.^4,22,23^ The difference between these approaches is therefore that the presence of the target condition is identified by the results of both tests in the composite reference standard approach, but on a mixture of reference test results in discrepant analysis. A discrepant analysis is generally not recommended since it entails a large bias. This bias can stem from the consideration of the index test result in the definition of true target condition status,^23,24^ the correlation between the first reference test and the index test, and multiple misclassifications.^23^

Instead of applying pre-defined decision rules for a composite reference standard, an expert panel can be established that decides on the presence of the target condition by taking into account several sources of information (reference standard test results, participants’ characteristics and symptoms, follow-up information, and response to treatment).^4,23,24^ Different decision processes are possible: each expert decides independently, discrepant decisions are discussed until a consensus or majority vote, or experts discuss together to reach a consensus for each case.^23^ Employing an expert panel is an adequate approach if the target condition is only vaguely defined, can produce a wide range of possible symptoms,^23^ or if there is no accepted reference standard so far.^4^ Hitherto, there are few recommendations on the practical execution of a panel decision process. Thus, accuracy estimates of the index test can differ according to the characteristics of this process: the information on which the experts decide on the presence of the target condition, the decision process, the high influence of “strong personalities” in consensus meetings, and poor inter-rater agreement. In addition, the experts’ decision can be affected by the knowledge of index test results which can lead to an incorporation bias.^23,24^

Another option is the use of latent class models (LCMs) in the case of several imperfect tests for the target condition.^4,23,24^ LCMs consider the true target condition status as a latent variable (unknown) and estimate it by using the present imperfect reference and index tests as manifest variables. While basic LCMs assume conditional independence, there are also some more advanced LCMs that work under conditional dependence.^23^ Overall, LCMs are flexible as they can be applied for categorical as well as continuous tests and more than three tests. Nevertheless, they need information from at least three tests to be identifiable and may produce overestimated accuracy estimates if conditional dependence is not adequately considered.^4,23^ Accuracy estimates obtained in LCM are also rather statistically defined and not based on clinical characteristics.^23,24^ One way to improve the robustness and identifiability of the model is to incorporate prior information, that is, conducting a Bayesian LCM.^4,23,26^ As a prerequisite, prior information must be available in the literature or from experts. On the other hand, accuracy estimates are influenced by the chosen prior distribution and require more programming expertise.^23^

A final option may be to conduct a validation study that investigates the ability of the index test to predict the clinical event under study compared with a reference test.^4,23,24^ However, a validation study does not provide information on standard accuracy measures such as sensitivity or specificity as a diagnostic study.

To facilitate the choice for one of the presented approaches, Reitsma et al.^24^ provided a flowchart guiding the decision based on the availability of one or multiple imperfect reference standards. Additionally, flowcharts for choosing a specific method within an overall approach depending on index test characteristics and missingness mechanism can be found by Chikere et al.^4^ Besides articles included in the presented reviews and the reviews themselves, we could also identify articles (*n*  =  46) on handling missing values in the reference standard or an imperfect reference standard that were not included in the presented reviews. An overview of these additional articles and their classification into the main approaches described by Chikere et al.^4^ is available in the Supplemental Material.

### 4.2 Missing values in the index test

Several methods have been proposed across 15 articles to address missing values in the index test in scenarios where all individuals are verified with a reference test. These can be classified into imputation methods, frequentist, and Bayesian likelihood approaches as well as model-based approaches (see Table 1 and Supplemental Figure S1).

**Table 1.** Classification of approaches to handle missing values in the index test, in the index test and reference standard or inconclusive results.

Approach	Examples of specific methods	References
*Missing values in the index test*		
Imputation - single imputation	Single imputation: worst case scenario, disease prevalence imputation, imputation according to sensitivity/specificity or non-informative imputation, Random hot deck	Schuetz et al.,^11^ Campbell et al.,^22^ and An^27^
- multiple imputation	Multiple imputation	Cheng and Tang,^28^ Long et al.,^29^ and Gad et al.^30^
Frequentist maximum likelihood approaches	Independent case analysis, available independent case analysis, hybrid approach (maximum likelihood plus weighted least squares)	Poleto et al.^21^
Bayesian likelihood approaches	Bayesian adoption of the hybrid approach; Bayesian empirical likelihood approach;	Paulino and Silva^31^ and Lin et al.^32^
Generalized estimating equations	Marginal model for repeated measurements (comparison of several index tests across studies in a meta-analysis), dependent independent case analysis	Poleto et al.^21^ and Siadaty et al.^33^
Model-based	Inverse probability weighting, inverse probability weighting plus Regression models	Bianco et al.^34^ and Long et al.^35^
Comparing partially paired data		Zhou and Gatsonis^36^ and Martinez-Camblor^37^
*Missing values in the index test and reference standard*		
Model-based	Inverse probability weighting	Yu et al.^38^
Frequentist likelihood approaches	Maximum likelihood double verification model; ROCKIT	van Geloven et al.^39^ and Metz et al.^40^
Bayesian likelihood approaches	Bayesian hierarchical network meta-analysis model	Ma et al.^14^ and Lian et al.^41^
Latent class models	Different latent class models considering conditional dependence or not are available	Zhang^42^ and Zhang et al.^43^
Imputation	Different multiple imputation models Random hot deck imputation	Karakaya et al.^44^ Yang and Zhao,^45^ Wang and Qin,^46^ Wang and Qin,^47^ and Liu and Zhao^48^
*Inconclusive test results*		
Calculation of additional parameters	Calculating conditional sensitivity/specificity (by excluding inconclusive results) and giving additional parameters (e.g. test yield), a combined approach of Simel et al. (1987) and Begg and Greenes (1983) method	Simel et al.^8^ El Chamieh et al.^49^
Imputation - single imputation **- multiple imputation**	Worst case scenario, positive/negative imputation; intention-to-diagnose	Shinkins et al.^10^ and Schuetz et al.^11^ El Chamieh et al.^49^
Frequentist likelihood approaches	Extended trivariate generalized linear mixed model, trivariate/quadrivariate copula mixed model	Ma et al.^20^ and Nikoloulopoulos^50,51^
Bayesian likelihood approaches	Bayesian random effects model for intention-to-treat	Menke and Kowalski^52^
Defining and excluding an area of inconclusive results	Two-graph-ROC method, grey zone method, uncertain interval	Greiner,^53^ Landsheer,^54^ Landsheer,^55^ Coste et al.,^56^ and Coste and Pouchot^57^
Latent class model	Different latent class models considering conditional dependence or not are available	Xu et al.^58^

#### 4.2.1 Imputation methods

Both single imputation and MI approaches were proposed to address missing values in the index test. These single imputation methods include, for instance, the intention-to-diagnose approach (missing values are considered negative for the subject with the target condition and positive for those without the target condition, also referred to as worst-case scenario),^22^ random hot deck imputation^27^ or positive or negative imputation.^22^ In general, single imputation approaches are easy to implement, can provide insights into the accuracy under different assumptions (e.g. by providing the lower bounds of sensitivity/specificity under worst case/intention-to-diagnose),^22^ and are more efficient than complete case analysis. However, they are highly likely to give biased accuracy estimates in many scenarios and to distort the distribution of the data and correlation between variables if the imputation model does not reflect the missingness pattern. In addition, they often lead to an underestimation of variability.^59^ This applies particularly to the worst-case scenario, positive, and negative imputation.^20^

To overcome these limitations, MI has been proposed.^28–30^ MI is a quite flexible approach, which can include auxiliary variables that are associated with the missing values but not included in the analysis model, and estimates the variability more accurately. Commonly, MI assumes a multivariate normal distribution of the data, and its estimates are, therefore, sensitive to model misspecification. However, non-parametric imputation models (such as a k-nearest neighbor) can also be combined with the MI approach.^29^ Nonetheless, its flexibility, the different ways to build an imputation model, or the choice of variables to include in the imputation model, for example, makes the implementation of MI more complex. While MI methods can deal with data that are MAR (including MCAR), most single imputation strategies model an (often not justified) MNAR mechanism (e.g. positive/negative imputation and intention-to-diagnose) or are only applicable under MCAR.

#### 4.2.2 Likelihood approaches

Besides imputation methods, frequentist maximum likelihood^21^ and Bayesian maximum and empirical likelihood approaches^31,32^ can be applied. Maximum likelihood can be simpler to implement than MI as it does not require so many decisions regarding the model-building process. Nonetheless, several index tests are often required to achieve identifiability if several parameters need to be estimated and data must be parametric.^21^ To overcome the problem of possible non-identifiability, prior information can be included following a Bayesian approach. Such Bayesian likelihood approaches are more flexible in the number of parameters to be estimated.^32^ By including priors, the model becomes sensitive to the chosen prior distribution. This may result in estimates that strongly reflect the prior distribution in small samples.^31^ The proposed likelihood-based methods can handle missing values that are MAR.

#### 4.2.3 Other model-based approaches

Furthermore, Bianco et al.^34^ and Long et al.^35^ presented approaches based on regression models, such as inverse probability weighting which weights the observed values by the inverse probability of being missing given the true target condition status and possible covariates. As these model-based approaches are biased if the model is misspecified, Long et al.^35^ extended their method to include a second model based on a prediction score in addition to inverse probability weighting to develop a double robust approach. Most of these model-based approaches are valid under MAR but the doubly robust method by Long et al.^35^ can also include a parameter to model a MNAR scenario.

### 4.3 Missing values in the index test and reference standard

In addition to the presented methods addressing missing values in the index test (but assuming full verification), several articles (*n*  =  12) described strategies to handle missing values in both the index test as well as the reference standard (Table 1 and Supplemental Figure S2). In line with the strategies described above, these methods include single^45–48^ and MI methods,^44^ frequentists,^39,40^ and Bayesian likelihood^14^ approaches, and inverse probability weighting.^38^ Moreover, latent class methods (LCMs) have been proposed which consider the (imperfect) reference standard as a latent variable.^42,43^ Thus, the target condition status is statistically defined by the model and not by clinical criteria. Similarly to maximum likelihood approaches, LCMs require several tests to achieve the identifiability of the model.^42^ One important issue to be considered is conditional dependence between the test results included in the model. As assuming conditional independence is often not appropriate, Zhang^42^ also developed an LCM under the conditional dependence assumption. Consequently, the model implementation becomes quite complex and computationally expensive. In general, different LCM result in different accuracy parameters and it is often difficult to decide on the right model.

While the proposed single imputation methods are only applicable to MCAR, the Bayesian, as well as MI and inverse probability weighting approach and LCM can be used for MAR data. The Bayesian approach and LCM can, in addition, be extended to model MNAR. The ML double verification method can be employed for missing values in the reference test under MAR and MNAR but only for missing index test values under MCAR.

### 4.4 Handling inconclusive results

Before selecting an adequate analysis method, it is essential to pay attention to a correct and transparent display of the test results. Regarding a categorical index test, Simel et al.^8^ have already proposed the 6-cell matrix which displays inconclusive results of the index test stratified by true target condition status in separate cells. Moreover, Shinkins et al.^10^ specify that valid inconclusive results should be included in this 6-cell matrix while invalid inconclusive results and missing values should be presented in a separate table stratified by true target condition status. The 6-cell matrix was extended by Schuetz et al.^11^ to a 9-cell matrix (3  ×  3 table) if there are inconclusive results in both the index test and the reference test (adding a column for inconclusive results in the reference test).

Most identified articles (*n*  =  17, one of them also addressed missing values in the index test and is, therefore, included in both sections) focused on inconclusive results in general without distinguishing between uninterpretable, intermediate or indeterminate, or different patterns of inconclusiveness. Methods for the analysis of inconclusive results encompass simple methods, namely single imputation, and more complex methods, particularly frequentist and Bayesian likelihood approaches and LCM (see Table 1 and Supplemental Figure S3).

The single imputation models proposed in the context of inconclusive results are comparable to those regarding missing values (intention-to-diagnose, positive/negative imputation).^10,11^ In addition to calculating conditional sensitivity and specificity (conditional on a positive or negative result, excluding inconclusive test results), Simel et al.^8^ further recommended calculating additional parameters, such as the test yield, which describes the probability of obtaining either a positive or negative results of all possible test results and should be interpreted in line with sensitivity and specificity. Furthermore, the odds ratio of obtaining an inconclusive result in individuals with the target condition compared to those without the target condition can be computed.^8^ However, clinical physicians may not be accustomed to these new parameters which may bear the risk of neglecting them in the interpretation of test accuracy. El Chamieh et al.^49^ expanded Simel et al.'s^8^ approach by Begg and Greene's^60^ method to address cases where the inconclusive results in the index test and missing values in the reference standard are present. (El Chamieh et al.^49^ also used MI for handling inconclusive results, but they did not provide a detailed discussion of the performance.) The Bayesian approach developed by Menke and Kowalski^52^ follows the intention-to-diagnose approach, and the recommendations by Simel et al.^8^ and is applied to a meta-analysis where data on diagnostic tests from several samples are pooled. Likewise, the frequentist likelihood approaches^20,50,51^ are developed for meta-analyses and are quite sophisticated.

If imperfect reference standards and inconclusive results in the index test are present, an LCM may be used for the analysis. Xu et al.^58^ showed an extended log-linear and a probit LCM which considers inconclusive results and conditional dependence. Furthermore, there are three methods that aim at defining an interval of inconclusive—specifically intermediate—results in a continuous index test: two-graph-ROC,^53,54^ grey zone method,^54,57^ and the uncertain interval.^54,55^ This interval of inconclusive results is then excluded from the calculation of accuracy parameters leading to a reduction in power but also to higher accuracy parameters (since the intermediate values are excluded). Consequently, these accuracy parameters are only valid for the index test values which are above or below the identified interval. In clinical practice, it means that if patients have an index test value within this interval, no conclusion about the presence or absence of the target condition can be made and, as a result, they must undergo a second test or repeat this test after a defined time (depending on the target condition under question and clinical implications).^54^ Nonetheless, it must be made transparent in the reporting of the diagnostic study for which the range of values and the calculated accuracy parameters are valid. Otherwise, the performance of the test is heavily overestimated which may encourage incorrect or suboptimal clinical decisions.

## 5 Discussion

In summary, our literature search revealed that there are many strategies to handle missing values in the reference standard or an imperfect reference standard which have already been compiled and presented by some reviews.^4,23,24^ Therefore, the focus of our review was on providing an overview of methods to handle missing values in the index test and inconclusive results in diagnostic studies. Although an initial unstructured literature search at the beginning of this project indicated a lack of methods for handling missing values and inconclusive results in the index test, our scoping review could, in fact, identify several strategies which have been proposed for this issue. Regarding missing values, these methods comprised single imputation and MI, frequentist and Bayesian likelihood approaches, model-based strategies, and LCM. Most of these methods can be applied under MCAR and MAR. There are, still, only a few that can model MNAR. Methods that address inconclusive results similarly include single imputation, frequentist, and Bayesian likelihood approaches, and, in addition, an approach to defining an interval of inconclusive results in a continuous index test which is then excluded from the analysis.

In classifying the identified methods, it became evident that little consensus exists in the literature about coherent terminology of inconclusive results. Whereas the definition of missing values is mostly clear (i.e. no test result has been recorded), inconclusive test results can be distinguished into uninterpretable, intermediate, and indeterminate.^10^ According to Shinkins et al.,^10^ uninterpretable test results should be handled similarly to missing values as they give no information on the test value. Intermediate and indeterminate results, however, give information on the index test value. Nonetheless, it is not always clear which type of inconclusive results is handled by a specific method. Some articles did not use this terminology at all and spoke of non-evaluable results without giving a clear definition of non-evaluable.^20,50–52^ In general, we would encourage researchers to clearly indicate the type of inconclusive result or whether they speak of missing values. In line with Shinkins et al.,^10^ we propose to address missing values and invalid inconclusive results (uninterpretable) similarly in the statistical analysis by employing methods listed in Sections 4.2 and 4.3. Additionally, methods from Section 4.4 referring to non-evaluable results can also be used: the frequentist and Bayesian likelihood approaches by Mai et al.,^20^ Nikoloulopoulos,^50,51^ and Menke and Kowalski.^52^ Valid inconclusive results should be handled differently by using the remaining methods described under Section 4.4.

For the reporting of diagnostic studies in general, we would like to refer to recommendations by Simel et al.,^8^ Schuetz et al.,^11^ and Shinkins et al.^10^ who propose to present not only a 4-cell matrix but better a 6 or 9-cell matrix including inconclusive results as a separate category. It may additionally be useful to provide a table or flowchart of all (known) causes for missing values, indeterminate, intermediate, and uninterpretable results. Shinkins et al.^10^ further highlighted including valid inconclusive in the 6-cell matrix and constructing a separate table that shows invalid inconclusive results and missing values stratified by true target condition status. Regardless of which presentation of results is preferred, the number of missing values and inconclusive results and their handling in the analysis must be transparently reported—as it is recommended in the STARD guideline and defined in the STARD flow diagram.^2^

Although we could identify a range of methods addressing missing values and inconclusive results in the index test, they have hardly been applied in practice (see Supplemental Material for references of clinical application). Commonly, simple single imputation methods are employed or missing values/inconclusive results are excluded from the analysis.^10,11^ One reason might be that reporting guidelines such as STARD^2^ and QUADAS^61^ focus on encouraging transparent and comprehensive reporting in diagnostic studies and miss to emphasize the importance of dealing with missing values and inconclusive results appropriately in the analysis. Despite pointing out that bias can result from the exclusion of missing values, the STARD guideline merely lists some simple imputation methods without giving a clear recommendation for their use or without indicating the need for a more complex methodology.^2^ Additionally, the lack of application of these methods may be due to difficulties to find them. In fact, most of the articles that proposed methods for missing values and inconclusive results were identified through citation searching and not through our search strategy. Naturally, this may also be a limitation of our search strategy but it also illustrated how difficult and time-consuming it may be for other researchers to find the right method for their diagnostic study. Furthermore, only a few articles provide their analysis code (*n*  =  7), an R package, or other software (*n*  =  5) that facilitates the application of the proposed method for other researchers. In contrast, most of the identified articles about missing values in the index test do not provide the source code, or refer to R packages or websites that have been deleted from the repository or are not valid anymore. The only valid and up-to-date R packages, that were applied in some identified studies, are mice,^62^ mi,^63^ mix,^64^ and CopulaREMADA.^65^ In our Supplemental Material under the respective reference, we indicate for each method whether some implementation tool is available and, if so, where it can be found. The provision of tools becomes particularly relevant against the background that many of these methods—especially the LCM as well as the Bayesian and some of the frequentist likelihood approaches—are quite sophisticated. It may be difficult for clinicians to transfer the theoretical description of the method to its implementation in their diagnostic study. Hitherto, there has also not been any systematic overview and comparison of the identified methods which may be a reason for widespread uncertainty on adequate methodology for missing values and inconclusive results. By providing an overview of such methods, this position paper closes this gap and might enhance the visibility of the identified methods as well as the necessity to address missing values and inconclusive results properly in the analysis of diagnostic studies.

In our Supplemental Material, all methods are described in detail including advantages and disadvantages, missingness patterns, clinical application (if present), and indication if specialized software of syntax for statistical packages are available. Nonetheless, it must be emphasized that it may not always be necessary to use a sophisticated method for handling missing values or inconclusive results. If the share of missing values is very low, excluding them from the analysis will usually not have a large impact on the parameter estimate. Nevertheless, Newman^66^ discourages the use of complete case analysis as it “would be suboptimal, potentially unethical, and totally unnecessary.” Even with only a small proportion of missing values, the power is reduced and results may be biased as they are only transferable to participants who have answered the survey completely.^66^ Instead, pairwise deletion or single imputation methods may be an option if the partial missingness rate (the share of participants with construct-level missingness (not item-level missingness) for at least one construct out of all respondents) is below 10%. Still, this cut-off is arbitrary and must be applied with caution.^60^ In choosing a suitable analysis method, it must be kept in mind, that different methods may also measure slightly different parameters. This can be due to the fact that they have, for instance, different assumptions or are applied under different missingness patterns. In general, it can be recommended to conduct sensitivity analyses to examine the robustness of the results, especially if the proportion of missing values or inconclusive results is high. At this point, we cannot make concrete recommendations about which of the identified method would be the “most adequate” method for a certain scenario, since our primary aim was to identify and compile methods for handling missing values in diagnostic studies. Future research must focus on the systematic comparison of those methods in order to make reasonable recommendations for their use. Naturally, it is emphasized that possible measures to prevent the occurrence of missing values and inconclusive results should be taken in planning the study. In this planning process, possible reasons for (unavoidable) missing values and inconclusive results should be anticipated and an approach for handling them should be determined a priori (in a study protocol for instance).^7^ This can be illustrated in a special case of diagnostic studies using registry or hospital data. Depending on the aim of the study, researchers must decide whether they either include only subjects who received both the index test and reference standard or also those who missed one of both results. The target population and, consequently the calculated estimates, may differ between both approaches.

### 5.1 Limitations and strengths

Finally, some limitations of our review must be mentioned. Despite being quite extensive, our search strategy missed relevant articles which were identified through citation searching. Hence, there may still be methods that were also not discovered through citation searching and are, thus, not included in our review. Furthermore, there may be methodological papers that have been published after our search in April 2022 or in another language than English or German. As our review aimed to give a first overview of strategies to handle missing values and inconclusive results in diagnostic studies, the application and comparison of the performance of the described methods were beside the scope of this article. Future research is needed to compare the performance of these methods under different scenarios to give evidence-based recommendations for selecting the most optimal approach for a given scenario.

Nonetheless, our review has several strengths worth noting. To our knowledge, we present the first review of methods addressing missing values or inconclusive results in the index test. Moreover, we provide a detailed summary and description of the proposed methods in the Supplemental Material. At last, we searched several databases using an extensive search strategy and complemented this search with thorough citation searching.

## 6 Conclusion

In summary, our position paper gives an overview of strategies to handle missing values, inconclusive results in the reference standard or index test, and an imperfect reference standard. Despite the availability of a range of methods for missing values and inconclusive results in the index test, these strategies have hardly been applied in practice. This article contributes to enhancing the visibility of the identified methods and provides support for analysts of diagnostic studies in deciding on the appropriate method. Nevertheless, there is a need for further research that compares and evaluates the performance of the identified methods under various scenarios. Moreover, it must be emphasized that researchers developing a new method should provide an analysis code, R package, or a Shiny app to encourage an easy application of their method by other researchers.

## Supplemental Material

sj-docx-1-smm-10.1177_09622802231192954 - Supplemental material for Missing values and inconclusive results in diagnostic studies – A scoping review of methods Click here for additional data file. Supplemental material, sj-docx-1-smm-10.1177_09622802231192954 for Missing values and inconclusive results in diagnostic studies – A scoping review of methods by Katharina Stahlmann, Johannes B Reitsma and Antonia Zapf in Statistical Methods in Medical Research

sj-docx-2-smm-10.1177_09622802231192954 - Supplemental material for Missing values and inconclusive results in diagnostic studies – A scoping review of methods Click here for additional data file. Supplemental material, sj-docx-2-smm-10.1177_09622802231192954 for Missing values and inconclusive results in diagnostic studies – A scoping review of methods by Katharina Stahlmann, Johannes B Reitsma and Antonia Zapf in Statistical Methods in Medical Research

sj-tif-3-smm-10.1177_09622802231192954 - Supplemental material for Missing values and inconclusive results in diagnostic studies – A scoping review of methods Click here for additional data file. Supplemental material, sj-tif-3-smm-10.1177_09622802231192954 for Missing values and inconclusive results in diagnostic studies – A scoping review of methods by Katharina Stahlmann, Johannes B Reitsma and Antonia Zapf in Statistical Methods in Medical Research

sj-tif-4-smm-10.1177_09622802231192954 - Supplemental material for Missing values and inconclusive results in diagnostic studies – A scoping review of methods Click here for additional data file. Supplemental material, sj-tif-4-smm-10.1177_09622802231192954 for Missing values and inconclusive results in diagnostic studies – A scoping review of methods by Katharina Stahlmann, Johannes B Reitsma and Antonia Zapf in Statistical Methods in Medical Research

sj-tif-5-smm-10.1177_09622802231192954 - Supplemental material for Missing values and inconclusive results in diagnostic studies – A scoping review of methods Click here for additional data file. Supplemental material, sj-tif-5-smm-10.1177_09622802231192954 for Missing values and inconclusive results in diagnostic studies – A scoping review of methods by Katharina Stahlmann, Johannes B Reitsma and Antonia Zapf in Statistical Methods in Medical Research
