# Supplementary material for: Missing values and inconclusive results in diagnostic studies – A scoping review of methods
Source: Stat Methods Med Res. 2023 Aug 9;32(9):1842–55. doi: 10.1177/09622802231192954 (PMC10540494; doi:10.1177/09622802231192954)
Supplement: sj-docx-1-smm-10.1177_09622802231192954 - Supplemental material for Missing values and inconclusive results in diagnostic studies – A scoping review of methods [file sj-docx-1-smm-10.1177_09622802231192954.docx]

**Supplemental Material: Missing values and inconclusive results in diagnostic studies – a scoping review of methods**

**Content**

1. The search strategy
2. References excluded in the full-text screening and reasons for exclusion
3. Overview of strategies to handle missing values or inconclusive results in diagnostic studies
4. Full list of references included in this scoping review
5. References listed under clinical application

**List of Abbreviations**

AICA Assumed Independent Case Analysis

AUC Area under the Receiver Operating Characteristic Curve

BEL Bayesian Empirical Likelihood Approach

CCA Complete Case Analysis

CI Conditional Independence

CD Conditional Dependence

DICA Dependent Independent Case Analysis

GEE Generalized Estimating Equations

ICA Independent Case Analysis

IHBEL Imputation-based hybrid bootstrap empirical likelihood

IPEL Imputation-based empirical likelihood

IPW Inverse Probability Weighting

JEL Jackknife Empirical Likelihood

LCM Latent Class Modelling

LLLC Log-Linear Latent Class Model

LR Likelihood Ratio

MAR Missing at Random

MCAR Missing Completely at Random

MI Multiple Imputation

ML(E) Maximum Likelihood (Estimator)

n.a. Not applicable

MNAR Missing not at Random

NPV Negative Predictive Value

OR Odds Ratio

PLC Probit Latent Class Model

PPV Positive Predicted Value

PSM Propensity Score Model

(R)MSE (Root) Mean Squared Error

ROC Receiver Operating Characteristic Curve

SD Standard Deviation

SE Standard Error

TGLMM Trivariate Generalized Linear Mixed Model

TG-ROC Two Graph Receiver Operating Characteristic Curve

WLS Weighted Least Square

1. **The search strategy**

Medline/PubMed:

(diagnostic [title/abstract]) AND (“missing value*” [title/abstract] OR “missing data” [title/abstract] OR “incomplete data” [title/abstract] OR inconclusive [title] OR indeterminate [title] OR intermediate [title] OR uninterpretable [title] OR “without gold standard” [title/abstract] OR "missing gold standard" [title/abstract] OR "imperfect reference standard" [title/abstract] OR "imperfect reference test" [title/abstract] OR "no reference standard" [title/abstract] OR "missing reference standard" [title/abstract] OR "partial verification" [title/abstract] OR "differential verification" [title/abstract] OR “without index test” [title/abstract] OR “missing index test” [title/abstract] OR “no index test” [title/abstract] OR “missing diagnostic test” [title/abstract] OR “no diagnostic test” [title/abstract] OR “without diagnostic test” [title/abstract] OR “no comparator test” [title/abstract] OR “without comparator test” [title/abstract] OR “missing comparator test” [title/abstract])

- 2,176 results (April 13, 2022), (4 Duplicates)

Cochrane Library (including reviews, trials, and editorials):

diagnostic:ti,ab AND ((“missing value*” OR “missing data” OR “incomplete data” OR “without gold standard” OR "missing gold standard" OR "imperfect reference standard" OR "imperfect reference test" OR "no reference standard" OR "missing reference standard" OR "partial verification" OR "differential verification" OR “without index test” OR “missing index test” OR “no index test” OR “missing diagnostic test” OR “no diagnostic test” OR “without diagnostic test” OR “no comparator test” OR “without comparator test” OR “missing comparator test”):ti,ab OR (inconclusive OR indeterminate OR intermediate OR uninterpretable):ti)

- 326 results (April 27, 2022), (104 duplicates with MEDLINE results)

Web of Science:

(((TI=(diagnostic)) OR (AB=(diagnostic))) AND ((AB=(“missing value*” OR “missing data” OR “incomplete data” OR “without gold standard” OR "missing gold standard" OR "imperfect reference standard" OR "imperfect reference test" OR "no reference standard" OR "missing reference standard" OR "partial verification" OR "differential verification" OR “without index test” OR “missing index test” OR “no index test” OR “missing diagnostic test” OR “no diagnostic test” OR “without diagnostic test” OR “no comparator test” OR “without comparator test” OR “missing comparator test”)) OR (TI=(“missing value*” OR “missing data” OR “incomplete data” OR “without gold standard” OR "missing gold standard" OR "imperfect reference standard" OR "imperfect reference test" OR "no reference standard" OR "missing reference standard" OR "partial verification" OR "differential verification" OR “without index test” OR “missing index test” OR “no index test” OR “missing diagnostic test” OR “no diagnostic test” OR “without diagnostic test” OR “no comparator test” OR “without comparator test” OR “missing comparator test” OR inconclusive OR indeterminate OR intermediate OR uninterpretable))))

- 3,824 results (April 28, 2022), (2134 duplicates with MEDLINE & Cochrane results)

1. **References excluded in the full-text screening and reasons for exclusion**

| Autor(s) | Year | Database | Reason for exclusion |
| --- | --- | --- | --- |
| Aron et al. | 2021 | PubMed | no diagnostic accuracy study |
| Askarian et al. | 2016 | WoS | no diagnostic accuracy study |
| Biswas | 2016 | PubMed | no discussion of methods |
| Campbell & Yue | 2016 | PubMed | no discussion of methods |
| Chaouch et al. | 2014 | PubMed | no discussion of methods |
| Choi et al. | 2018 | PubMed | no diagnostic accuracy study |
| Dai et al. | 2018 | PubMed | no diagnostic accuracy study |
| de Groot et al. | 2014 | PubMed | language |
| Dimou et al. | 2010 | PubMed | no diagnostic accuracy study |
| Emerson et al. | 2018 | PubMed | no discussion of methods |
| Ensor et al. | 2018 | PubMed | no common outcome parameter |
| Fernandez-Alonzo et al. | 2012 | PubMed | language |
| Ferreira-Santos et al. | 2018 | PubMed | no diagnostic accuracy study |
| Glueck et al. | 2009 | PubMed | no discussion of methods |
| Holtman et al. | 2013 | PubMed | no discussion of methods |
| Jolani et al. | 2015 | PubMed | no diagnostic accuracy study |
| Kolehmainen et al. | 2006 | PubMed | no diagnostic accuracy study |
| Li et al. | 2019 | WoS | no diagnostic accuracy study |
| López Pineda et al. | 2015 | PubMed | no diagnostic accuracy study |
| Lou et al. | 2018 | WoS | no diagnostic accuracy study |
| Martín-Baranera et al. | 2000 | PubMed | no diagnostic accuracy study |
| Matos et al. | 2011 | PubMed | no discussion of methods |
| Roldan-Nofuentes & del Castillo | 2006 | WoS | no common outcome parameter |
| Roldan-Nofuentes & del Castillo | 2007 | WoS | no common outcome parameter |
| Poirier & Ruud | 1983 | WoS | no diagnostic accuracy study |
| Ringham et al. | 2010 | PubMed | no discussion of methods |
| Rodriguez-Alvarez et al. | 2016 | PubMed | no diagnostic accuracy study |
| Roldan Nofuentes et al. | 2014 | WoS | no common outcome parameter |
| Roldan Nofuentes & Regad | 2021 | WoS | no common outcome parameter |
| Roldan Nofuentes & Regad | 2021 | WoS | no common outcome parameter |
| Roldan Nofuentes & Regad | 2020 | WoS | no common outcome parameter |
| Santos et al. | 2018 | PubMed | no discussion of methods |
| Shan & Wang | 2020 | PubMed | no diagnostic accuracy study |
| Song et al. | 2022 | WoS | language |
| Thung er al. | 2017 | PubMed | no diagnostic accuracy study |
| Toft et al. | 2019 | PubMed | no discussion of methods |
| van der Heijden et al. | 2006 | PubMed | no diagnostic accuracy study |
| Venkatraman et al. | 2016 | WoS | no diagnostic accuracy study |
| Viola et al. | 2020 | PubMed | no discussion of methods |
| Wahlstrom et al. | 2016 | WoS | no discussion of methods |
| Yu et al. | 2016 | PubMed | no discussion of methods |

1. **Overview of strategies to handle missing values or inconclusive results in diagnostic studies**
2. **Missing values in the reference standard or an imperfect reference standard**

Please see Reitsma et al. 2009, Rutjes et al. 2007, or Chikere et al. 2019 (especially their supplemental material) for a detailed review of several methods.

**Classification of articles that have not been included in the above-mentioned reviews**

| Application for | Approach | Author(s) | Year |
| --- | --- | --- | --- |
| Missing values in the reference standard | Correction | Arifin & Yusof | 2022 |
|  |  | de Groot et al. | 2012 |
|  |  | de Groot, Janssen et al. | 2011 |
|  |  | de Groot et al. | 2008 |
|  |  | Marin-Jimenez &  Roldan-Nofuentes | 2014 |
|  |  | Marin-Jimenez &  Roldan-Nofuentes | 2017 |
|  |  | Li & Gatsonis | 2019 |
|  |  | Lin et al. | 2006 |
|  |  | Ma et al. | 2016 |
|  |  | Montero-Alonso & Roldan-Nofuentes | 2019 |
|  |  | Pennello | 2011 |
|  |  | Roldan Nofuentes et al. | 2009 |
|  |  | Roldan Nofuentes & del Castillo | 2005 |
|  |  | Roldan Nofuentes | 2021 |
|  | Imputation | Cho et al. | 2019 |
|  |  | de Groot, Janssen et al. | 2011 |
|  |  | de Groot et al. | 2008 |
|  |  | Montero-Alonso & Roldan-Nofuentes | 2019 |
|  |  | Roldan Nofuentes | 2021 |
|  | LCA | Collins & Huynh | 2014 |
|  | Rasch Measurement Model | Cipriani et al. | 2005 |
|  | Review of several methods | Naaktgeboren et al. | 2016 |
|  |  | Unal & Burgut | 2014 |
| Imperfect reference standard | Correction | Coart et al. | 2015 |
|  |  | Enoe et al. | 2000 |
|  |  | Hahn et al. | 2019 |
|  |  | Schneeweiss | 2000 |
|  |  | Chikere et al. | 2021 |
|  | LCA | Asselineau et al. | 2018 |
|  |  | Boelaert et al. | 1999 |
|  |  | Collins & Albert | 2016 |
|  |  | Engel et al. | 2006 |
|  |  | García Barrado et al. | 2017 |
|  |  | Ghaemmaghami et al. | 2021 |
|  |  | Helman et al. | 2020 |
|  |  | Johnson et al. | 2019 |
|  |  | Liu et al. | 2015 |
|  |  | Liu et al. | 2022 |
|  |  | Mattner & Mattner | 2013 |
|  |  | McDonald & Hodgson | 2018 |
|  |  | Menten et al. | 2008 |
|  |  | Menten & Lesaffre | 2015 |
|  |  | Pence et al. | 2009 |
|  |  | Qu & Hadgu | 1998 |
|  |  | van Smeden et al. | 2014 |
|  |  | Wang et al. | 2020 |
|  |  | Cerullo et al. | 2022 |
|  | Neural networks | Walsh | 2018 |
|  | Machine learning | Wang et al. | 2013 |
| Missing values in the reference standard & an imperfect reference standard | Correction | Chen et al. | 2005 |

1. **Missing values in the index test**

Table 1. Missing values in **a single binary index test**

| **Method** | **Missing pattern** | **CI/**  **CD** | **Description** | **Strengths^a^** | **Weaknesses^a^** | **Reference(s)** |
| --- | --- | --- | --- | --- | --- | --- |
| *Single imputation* |  |  |  |  |  |  |
| Worst case scenario/Intention-to-diagnose | Any pattern | n.a. | Missing values are considered positive for non-diseased and negative for diseased patients.  *Clin. App. (examples): Lee et al. 2019; Nasis et al. 2010* | This strategy provides lower bounds of sensitivity and specificity. | It underestimates sensitivity and specificity; estimates are biased | Campbell et al. 2011  No packages mentioned nor code available. |
| Disease prevalence imputation | Any pattern | n.a. | Missing values are considered negative if disease prevalence is <0.5 |  | Specificity increases; accuracy parameters do not reflect the performance of the test itself anymore; estimates are often biased | Campbell et al. 2011  No packages mentioned nor code available. |
| Sensitivity/ Specificity imputation | MAR | n.a. | Missing values are considered positive (negative) proportionally to sensitivity (specificity) for the diseased and non-diseased patients without missing index test results, respectively. | Increases power compared to CCA; gives valid estimates under MAR; imputation can be performed multiple times (MI) to estimate adequate variance | This method can lead to biased parameter estimates if MCAR (or MNAR) is present. | Campbell et al. 2011  No packages mentioned nor code available. |
| Non-informative imputation | Anny pattern | n.a. | The proportion of missing values considered positive is based on the probability of observing a positive test result among all patients with valid test results | Sensitivity and specificity are shrunk relative to estimates from the completers analysis | Higher accuracy than worst case scenario, but gives conservative estimates | Campbell et al. 2011  No packages mentioned nor code available. |

^a^Strengths and Weaknesses are based on aspects which are mentioned by the authors of the respective method in their paper. In most cases, the proposed method has not been evaluated systematically by other studies. Hence, no other information on the performance of the respective method is available.

Table 2. Missing values in **several binary index tests**

| **Method** | **Missing pattern** | **CI/**  **CD** | **Description** | **Strengths^a^** | **Weaknesses^a^** | **Reference(s)** |
| --- | --- | --- | --- | --- | --- | --- |
| Independent Case Analysis (ICA) | MCAR | CI | Only those patients who have results on only one of the index tests (excluding those who have results for more than one index test or missing results for all index tests) are included in the analysis; the approach assumes product-multinominal distribution with each of two independent multinomial distributions corresponding to one of the index tests |  | The method ignores the dependence structure between index tests; biased results under MAR (MNAR); larger MSEs than CCA for some estimates; low power due to excluding patients with results of more than one index test | Poleto et al. 2011    The R package was removed from the CRAN repository, but can be accessed from the archive. The source code is online available: <http://www.poleto.com/missing.html?i=1>  [www.poleto.com/ACD.r](http://www.poleto.com/ACD.r) |
| Assumed Independent Case Analysis (AICA) | MCAR | CI | Patients with results for only one index test and with results on more than one index test are included (only those with missing results for all index tests are excluded); this approach ignores the dependence structure for those with more than one index test result; assumes product-multinominal distribution with each of two independent multinomial distributions corresponding to one of the index tests | Includes more observations 🡪 higher power; more efficient than CCA or ICA | Ignores dependence structure between index tests 🡪 larger and probably biased SEs; biased results under MAR (MNAR); | Poleto et al. 2011  The R package was removed from the CRAN repository, but can be accessed from the archive. The source code is online available: <http://www.poleto.com/missing.html?i=1>  [www.poleto.com/ACD.r](http://www.poleto.com/ACD.r) |
| Dependent Independent Case Analysis (DICA) | MCAR | CD | This method includes the same sample as AICA but considers the dependence structure; it applies a standard marginal analysis with adjustment for correlations with the sandwich variance estimator (e.g. the results of each diagnostic test, conditionally on true disease status, are assumed to have the mean-variance relation of the Bernoulli distribution (with parameter Sens or 1 − Spec) with an arbitrary “working" correlation matrix; to protect against misspecification, use the robust or empirically corrected covariance matrix); GEE method; same results as AICA under an independent working correlation | This method includes more observations than CCA or ICA 🡪 more efficient than CCA/ICA; it considers dependence structure | Excludes observations with missing values in all index tests; biased results under MAR (MNAR); | Poleto et al. 2011  The R package was removed from the CRAN repository, but can be accessed from the archive. The source code is online available: <http://www.poleto.com/missing.html?i=1>  [www.poleto.com/ACD.r](http://www.poleto.com/ACD.r) |
| Hybrid approach: ML and WLS | MCAR/ MAR | unclear | This method uses all observations (also those with missing values for all index tests); it defines an additional missingness indicator (W=1 if data on both index tests are present, W=2 result for only one index test is present, W=3 result for only the other index test is present, W=4 if data are missing for both); it considers the selection model factorization, where a marginal model for the measurements is combined with a conditional model for the missingness process given the measurements 🡪 model is not identifiable (over-parametrization), but constraints induced by assuming MAR; in case MCAR is valid WLS is conducted, for MAR ML | Valid under MAR; Estimates for sensitivity and specificity similar to methods above, but better approximation for the PPV and NPV (as disease prevalence is better estimated by including also those with data on true disease status but with missing values for all index tests); more efficient than approaches above as it uses all available data | This method might be extendable to a MNAR model, but this needs additional information, otherwise, it will not be identifiable | Poleto et al. 2011  The R package was removed from the CRAN repository, but can be accessed from the archive. The source code is online available: <http://www.poleto.com/missing.html?i=1>  [www.poleto.com/ACD.r](http://www.poleto.com/ACD.r) |
| Repeated-measurements meta-regression | unclear | CD | Meta-analysis of a range of index tests compared to different reference tests across studies; not all included studies examined the same index test; hence, there are missing values in the index tests across studies; assumption that the same reference test was used in all studies;  This method uses a marginal logistic model including covariates if desired and a variable indicating which reference standard was used for each study; applicable if no individual-level data are available but the 2x2 table for each included study  *Clin. app.: Ropka et al. 2010; Ropka et al. 2006; Philbrick et al. 2007; Virgili et al. 2007* | Predicted values, diagnostic odds ratios, and likelihood ratios are calculated; the method can be extended to use individual-level data; it accounts for the sample size of the individual studies | No direct calculation of sensitivity or specificity; does not consider misclassification or missing values in the reference standards | Siadaty et al. 2004  SAS code for some models in the appendix. URL link to website with R functions and more material is not valid anymore. |
| *Bayesian approach* |  |  |  |  |  |  |
| Bayesian adoption of the hybrid approach | MAR | unclear | Bayesian adoption of the hybrid approach by Poleto et al. (2011); the a priori distribution is a member of the Drichlet family (vague prior information), iterative Monte Carlo method based on the idea of chain data augmentation | This approach is useful for small samples with a high amount of missing data; incorporation of priors can improve the identifiability of the model | The computation based on Markov chain did not converge (hence chain data augmentation); sensitivity analyses necessary to investigate the effect of the prior distribution on accuracy estimates | Paulino & Silva 2019  No script for calculation is available. |
| *Imputation* |  |  |  |  |  |  |
| MI using chained equations | MAR | unclear | Here under MCAR studied (not MAR); the imputation model should include all variables needed for the analysis model (also the dependent variable and interaction terms); MICE package used for imputation; after imputation, accuracy estimates calculated by 1) simple proportion method, 2) logistic regression or 3) GEE | Also applicable under MAR; only small bias for all three analysis models; slightly higher MSE for GEE compared to the other two methods, but GEE has a slightly lower bias for some accuracy parameters; results of clinical application example similar to Poleto et al. (ML approach) | Building the imputation model may be complex | Gad et al. 2022  No code is available. They do not specify which package/command were used for the implementation of mice. However, mice is implemented in a range of softwares (e.g. mice R package). |

^a^Strengths and Weaknesses are based on aspects which are mentioned by the authors of the respective method in their paper. In most cases, the proposed method has not been evaluated systematically by other studies. Hence, no other information on the performance of the respective method is available.

Table 3. Missing values in a **single continuous index test**

| **Method** | **Missing pattern** | **CI/**  **CD** | **Description** | **Strengths^a^** | **Weaknesses^a^** | **Reference(s)** |
| --- | --- | --- | --- | --- | --- | --- |
| IPW approach to estimate ROC/AUC | MAR | n.a. | The probability of missingness conditional on covariates is modeled by logistic regression; the ROC/AUC are estimated by using IPW of this probability; this approach can be extended by adding a kernel-smoothing step | Good performance | Biased estimates when the propensity model is incorrect; convolution-based estimates are slightly better concerning MSE | Bianco et al. 2023  Programming code is not available. |
| Convolution-based ROC/AUC | MAR | n.a. | By assuming that the covariates are related to the index test through a regression model, convolution-based estimates can be calculated | Overall good performance; slightly better than IPW/IPW+kernel estimates regarding MSE | Biased estimates when regression function for convolution property is incorrect | Bianco et al. 2023  Programming code is not available. |
| IPW | MAR | n.a. | 1. PSM predicting missingness of the index test using auxiliary variables and true disease status  2. IPW using the PSM for calculating the AUC | Easy to calculate and straightforward | Biased if PSM is misspecified (e.g. under MNAR), not very efficient; plotting the ROC is not straightforward | Long et al. 2011a  Programming code is not available. |
| Doubly Robust estimation (parametric) 🡪 augmented IPW (AIPW) | MAR/MNAR | n.a. | Approach as above (IPW); plus conditional mean model of test given auxiliary variables and the true disease state; AUC is calculated | Extension to investigate MNAR; doubly robust method (performs better regarding bias and efficiency than other methods, especially if the conditional mean model is correct but also even slightly better if both models are misspecified); it can also handle categorical tests; easier applicable than non-parametric extension (below) | Substantial loss of efficiency if the IPW model is correct but the conditional mean model is misspecified; substantial bias under MNAR if the conditional mean model is misspecified; plotting the ROC is not straightforward | Long et al. 2011a  Programming code is not available. |
| Doubly Robust estimation (non-parametric) 🡪 non-parametric AIPW | MAR/MNAR | n.a. | Approach as above (IPW); plus non-parametric conditional mean model of test given auxiliary variables and the true disease state for calculation of the AUC; SEs are calculated using bootstrap | Extension to investigate MNAR; doubly robust method (performs better regarding bias and efficiency than other methods, especially if the conditional mean model is correct but also even slightly better if both models are misspecified); fairly robust to misspecified residual distribution for the index test values | Bootstrap calculation of SEs computational expensive and more susceptible to large and unstable weights; substantial bias under MNAR if the conditional mean model is misspecified; Substantial loss of efficiency if the IPW model is correct but the conditional mean model is misspecified; plotting the ROC is not straightforward | Long et al. 2011a  Programming code is not available. |
| *Bayesian approach* |  |  |  |  |  |  |
| Bayesian empirical likelihood approach (BEL) | MAR | n.a. | The BEL method employs the empirical likelihood using a MW two-sample U-statistic by treating it as a profile likelihood using a parametric augmentation technique. Further, Markov chain Monte Carlo (MCMC) methods can be used by deriving a Gibbs sampler scheme to draw posterior samples based on the proposed empirical likelihood; BEL uses a single and coherent joint likelihood function; extension for incorporating missing index results: inverse probability weighting using a parametric PSM estimating the probability of response given true disease status and collected auxiliary variables plus modeling an outcome regression model (test result=outcome); use of non-informative priors | It is flexible (can be adapted to handle more complicated problems based on iterative posterior sampling: e.g. multiple tests) and double robust regarding its approach to addressing missing values; compared with the frequentist double robust method by Long et al. (2011), the BEL approach performs similarly well regarding bias and RMSE and has a narrower interval; performance improves with increasing sample size; BEL is better than Long et al. (2011) when outcome model is misspecified; gain of proposed method if the sample size is small | Sensitivity analyses are needed to investigate the effect of the chosen priors | Lin et al. 2021  R code is provided as supplemental material. |
| *Imputation* |  |  |  |  |  |  |
| Non-parametric knn MI approach | MAR | n.a. | PSM with the probability of missing value given true disease state and auxiliary variables; then defining a distance between observations with missing values and those with observed values based on the PSM; select k nearest neighbors for observations with missing values; choose one of the k neighbors randomly and impute its observed value for the observation with missing value; repeat this imputation procedure several times; parameter estimates are calculated for each imputed dataset and then summarized by Rubin’s rules to obtain a pooled estimate | MI increases the power and improves the variance estimation; the inclusion of “noise” auxiliary variables has only a minimal impact on parameter estimates | Fully observed auxiliary variables that are associated with missingness are necessary; difficulties to identify similar observations (neighbors) when the number of auxiliary variables is high; the model produces an unsatisfactory coverage rate and moderate bias; performance deteriorates quickly when the dimension of auxiliary variables increases; with too many k neighbors performance decreases as they are not that similar anymore | Long et al. 2011b  Programming code is not available. |
| Non-parametric MI based on a prediction score | MAR | n.a. | The prediction model for observed index test values given true disease status and auxiliary variables is defined by a linear regression; then apply prediction score to all observations and define distance in the predicted values between observation with observed and missing index test values; choose one of several close neighbors to those with missing values based on the predicted values randomly and impute the observed index test values; imputation procedure multiple times; parameter estimates are calculated for each imputed dataset and then summarized to obtain a pooled estimate | Addresses the problem of too many auxiliary variables; the inclusion of “noise” auxiliary variables has only a minimal impact on parameter estimates | Fully observed auxiliary variables that are associated with missingness are necessary; dependence on a correctly specified working model to ensure consistency (may be incorrect because of missing auxiliary variables among others); if the working model is incorrect substantial bias and inadequate coverage rates | Long et al. 2011b  Programming code is not available. |
| Non-parametric MI using knn & prediction score | MAR | n.a. | Construct PSM and prediction score as above; construct a summary distance measure on both the PSM and the prediction score (both can be equally weighted or differently) and identify k nearest neighbors; impute the observed value of one randomly chosen neighbor; repeat imputation procedure multiple times; parameter estimates are calculated for each imputed dataset and then summarized to obtain a pooled estimate | addresses the dependence on the correctly specified working model -> only one of both working models must be correct -> double robust method (small bias if one is incorrect); minimal loss of efficiency due to non-parametric calculation compared to parametric calculation; if both models are incorrect still better performance than standard (non)parametric MI; weights for the scores can be altered to account for prior beliefs of the correctness of the working model | MI procedures fail to account for the uncertainty of the regression estimates of both working models -> MI procedure is improper; although the final parameter estimates are still consistent, the MI variances may be underestimated | Long et al. 2011b  Programming code is not available. |
| Non-parametric MI using knn & prediction score with bootstrap | MAR | n.a. | Bootstrap step at the start: draw several bootstrap samples with replacement from the original dataset; then perform the procedure as above (MI using PSM and prediction score); summarize pooled estimates from all bootstrap samples | MI procedure proper; the model produces SEs and coverage ranges that are closer to true values than other methods; small bias if one working model is incorrect -> doubly robust; if both models are incorrect still better performance than standard (non)parametric MI; the magnitude of improvement associated with using bootstrap is greater when mean structure is misspecified; weights for the scores can be altered to account for prior beliefs of the correctness of the working model | Computational extensive | Long et al. 2011b  Programming code is not available. |
| Kernel-assisted estimation equation imputation (KA) | MAR | n.a. | Kernel-assisted estimation equation imputation | good performance even under misspecified missingness mechanism, better than the non-parametric approaches by Long et al. 2011; advantages of estimating equations: incorporation of auxiliary information, considering the correlated structure of index test values, no assumption of the distribution of index test values | Worse performance with respect to bias, root mean square, interval width, and coverage probabilities than the IPL method below | Cheng & Tang 2019  R code is provided as supplemental material. |
| MI approach for estimating equations | MAR | n.a. | Impute missing value randomly drawn from the conditional distribution (index test value conditional on auxiliary information), k independent imputations (k=20 proposed), then define set of imputed estimating equations | good performance even under misspecified missingness mechanism, better than the non-parametric approaches by Long et al. 2011; advantages of estimating equations: incorporation of auxiliary information, considering the correlated structure of index test values, no assumption of the distribution of index test values | Worse performance with respect to bias, root mean square, interval width, and coverage probabilities than the IPL method below | Cheng & Tang 2019  R code is provided as supplemental material. |
| Hybrid imputation combining IPW & MI (IPL) | MAR | n.a. | Construct PSM using logistic regression or non-parametric equivalent (probability of missingness given auxiliary variables); include IPW based on the PSM in the definition of imputed estimating equations; using smoothed empirical likelihood to calculate ROC, CIs | good performance even under misspecified missingness mechanism, better than the non-parametric approaches by Long et al. 2011; better than both methods above with respect to bias, root mean square, interval width, and coverage probabilities; recommended for moderate or large sample sizes; advantages of estimating equations: incorporation of auxiliary information, considering the correlated structure of index test values, no assumption of the distribution of index test values |  | Cheng & Tang 2019  R code is provided as supplemental material. |
| Imputation-based smoothed EL approach | MCAR | n.a. | First random hot deck imputation then conducting a smoothed EL approach to estimate ROC | Good coverage | Performance decreases with increasing missingness proportion but is still good | An 2012  Programming code is not available. |

^a^Strengths and Weaknesses are based on aspects which are mentioned by the authors of the respective method in their paper. In most cases, the proposed method has not been evaluated systematically by other studies. Hence, no other information on the performance of the respective method is available.

Table 4. Missing values in **multiple continuous index tests**

| **Method** | **Missing pattern** | **CI/**  **CD** | **Description** | **Strengths^a^** | **Weaknesses^a^** | **Reference(s)** |
| --- | --- | --- | --- | --- | --- | --- |
| Non-parametric approach for comparing ≥2 AUCs with missing data in a paired design | MCAR | CI | Non-parametric approach assuming the diseased persons and the non-diseased persons are a random sample of the diseased and non-diseased patients in the study; (true) disease status known for all, but test results of either of two index tests missing for some; assuming that having both test results is independent of test results; corrected method for estimating the AUC using all data including those where results are only available for one index test  *Clin. app. (examples): Benedikt et al. 2017; Conant et al. 2019; Link et al. 2006* | more efficient than complete case analysis; for continuous and ordinal tests | independence  assumption: missingness depends on true disease status but not on the responses of the two diagnostic tests | Zhou & Gatsonis 1996  Programming code is not available. |
| Non-parametric approach for comparing ≥2 AUCs with missing data in a paired design | MCAR/ MAR | unclear | The method estimates non-parametric AUCs separately for paired data (no missing values) and unpaired data (missing values in 1 index test); then combine both AUCs into one AUC estimate; weights can be assigned to the AUC for paired and for unpaired data; the same approach for both index tests to compare both AUCs | Makes use of all observed information; weights for unpaired and paired AUC can be changed to obtain a more general estimator; can be extended to more than two index tests | AUC may be biased in the case of MAR (MNAR) | Martinez-Camblor et al. 2013  Programming code is not available. |
| Doubly robust inverse probability weighting | MAR | n.a.  (index test results are summarized into a single score) | *For prospective cohort studies*  missing values are handled by inverse probability weighting, where both a parametric (modeling the missing data process using a proportional hazards model) and semi-parametric (modeling the conditional distribution of the index tests using a location model) model are augmented; multiple index tests (e.g. biomarkers) are summarized to one score for the evaluation of their accuracy | Robust to misspecification of one of the missingness models (parametric or semi-parametric) | Good performance of this method if at least one of the models is correctly specified; better in regard to bias than CCA or simple IPW | Li & Ning 2015  R code is provided as supplemental material. |

1. **Missing values in the reference standard and the index test**

Table 5. Missing values in a **binary reference test** and **multiple binary index tests**

| **Method** | **Missing pattern** | **CI/**  **CD** | **Description** | **Strengths** | **Weaknesses** | **Reference(s)** |
| --- | --- | --- | --- | --- | --- | --- |
| IPW | MAR | n.a.  (in the example only one index test is examined) | IPW is based on covariates using a generalized logit model with the probability of missingness as a dependent variable (four categories: 1= reference test and index test result missing, 2= only reference test missing, 3= only index test missing, 4=neither is missing) and covariates as independent variables; (equals WGEE for a single diagnostic criterion & without missing values in the reference standard) | Good performance under MAR and MCAR | type I error rate is upwardly biased for small sample sizes; low power for small samples; no control for covariates, not able to handle missing data that do not follow the same pattern; the same pattern of missing data across different diagnostic test assumed | Yu et al. 2008  >they state: comparing multiple diagnostic tests, but in examples, they only use one reference test and an index test  Programming code is not available. |
| ML-based double verification model | Reference test: MAR  Index tests: MCAR | CI | This model includes a disease component (probability of disease given covariates) and a verification component for patients with different missingness patterns (probability of verification given index test results, disease status, and covariates/patient characteristics); the method models two index tests (and a reference test) simultaneously | joint analysis of both index tests does result in smaller RMSE for all measures, smaller bias, and better estimation of prevalence; improved model fit if patient characteristics are included; specificity estimates robust against various verification assumptions; this approach makes use of all data | Correlation between index tests not considered (in this study’s example maybe not relevant); Missing values in the index tests are considered MCAR; the model-based approach can be biased in case of model misspecification; computational extensive | Van Geloven et al. 2011  The authors state that the source code is available upon request. |
| ML-based double verification model with MNAR parameter | Reference test: MNAR  Index tests: MCAR | CI | The model above plus adding a disease parameter to the verification model (e.g. patients with the true disease are more likely to receive the reference tests) | Improved model fit compared to the model above; joint analysis of both index tests results in smaller RMSE for all measures, and smaller bias; the approach makes use of all data | Performance depends on MNAR assumption; sensitivity analyses should be conducted to explore different MNAR mechanisms; the correlation between index tests is not considered; missing values in index tests are considered MCAR | Van Geloven et al. 2011  The authors state that the source code is available upon request. |
| Bayesian hierarchical NMA-DT model (network meta-analysis diagnostic test) | MAR / MNAR | CI | Using a mixed effects model with multivariate random effects; perspective as if all studies were conducted in a multiple test comparison design and the outcomes of the not conducted tests are considered missing values; missingness is considered as independent of its sensitivity and specificity; fixed effects for sensitivity, specificity, prevalence; random effects are study specific effects of prevalence, sensitivity, specificity; the model assumes the same covariance matrix for all studies; it employs likelihood estimation and vague normal prior (here inverse Wishart) with mean 0 and variance 10 are assumed for the covariance matrix; the model can be adapted to model MNAR by including a model of missingness  >for ROC see: Qinshu Lian, James S. Hodges & Haitao Chu (2019) A Bayesian Hierarchical Summary Receiver Operating Characteristic Model for Network Meta-Analysis of Diagnostic Tests, Journal of the American Statistical Association, 114:527, 949-961  *Clin. app.: Hai et al. 2022* | The model accounts for heterogeneity  across studies and the complex correlation structure among multiple diagnostic tests; it incorporates studies with different designs and produces nearly unbiased estimates with small MSE; results are more biased under MAR and MNAR than under MCAR or with higher correlation; coverage probabilities are high but decrease under MNAR; the model is more efficient and less biased under MAR and MNAR and has higher coverage than under CCA | sensitivity analyses to the prior distribution necessary to evaluate the effect of the prior on the posterior estimates; the model relies on consistency assumption (tests would have been conducted consistently on subjects assigned and not assigned to the test; would be violated if e.g. one tests would be inappropriate for the study population of another study), conditional independence of index tests not always valid | Ma et al. 2018  Programming code is not available.  Lian et al. 2019  Programming code is not available. |
| LCM with EM-algorithm | MAR/ MNAR | CI | Here: only multiple index tests with missing values under study, there was no gold or reference standard examined;  LCM is performed with the EM-algorithm; Louis’s formula is used to obtain the observed information matrix of the MLE estimated by the EM;  Expansion to account for MNAR: assuming that the missing pattern differs between diseased and non-diseased patients; adding a parameter to the model above which describes the missing probability for the respective test when the subject has the disease (r1) and when the subject has not the disease (r0); the hypothesis that r1=r0 can be tested for each index test | Coverage probabilities are best under MCAR; slightly lower for prevalence and sensitivity under MAR but still good (mostly >95%); even for high missing probability; coverage probabilities are only slightly lower under MNAR (still above 94%); good performance when missingness depends on another test or (unobserved) true disease status | Conditional independence assumption; substantial loss of performance under MNAR when missingness depends on the missing value itself; lack of clinical definition of the disease (rather defined by statistical model) | Zhang 2013;  R package is presented in the text, but it cannot be found online.  Zhang et al. 2014  Programming code is not available. |
| PLC model with PX Monte Carlo EM-algorithm | MAR/ MNAR | CD | Here: only multiple index tests with missing values under study, there was no gold or reference standard examined;  The similarity between index tests (conditional dependence) is described by a Gaussian latent variable z which has a multivariate normal distribution conditional on true disease status; as the PLC model is non-identifiable, the variance-covariance matrix is restricted to a correlation matrix with all diagonal elements=1 and all off-diagonal elements [-1,1]; then applying PX-Monte-Carlo EM algorithm; estimates of a TLC model under conditional independence assumption, which was adapted to allow missing values, are used as starting values; starting values for the latent variable z arbitrarily set to 0.5; bootstrap method employed to estimate SE | Assumption of conditional dependence; good performance of bootstrap method; under MCAR & MAR PLC model shows unbiased estimates and good coverage probabilities (around 95%); robust to a high proportion of missing data when starting values are close to true values; the model can include covariates | Under MNAR coverage probabilities are worse (some <90%); computational challenge and intensive; estimates are sensitive to starting values; the number of tests must be ≥5 to ensure identifiability; lack of clinical definition of the disease (rather defined by statistical model) | Zhang 2013  R package is presented in the text, but it cannot be found online. |

^a^Strengths and Weaknesses are based on aspects which are mentioned by the authors of the respective method in their paper. In most cases, the proposed method has not been evaluated systematically by other studies. Hence, no other information on the performance of the respective method is available.

Table 6. Missing values in a **binary reference standard** and a **single continuous index test**

| **Method** | **Missing pattern** | **CI/**  **CD** | **Description** | **Strengths^a^** | **Weaknesses^a^** | **Reference(s)** |
| --- | --- | --- | --- | --- | --- | --- |
| *Imputation* |  |  |  |  |  |  |
| MI – different approaches | MAR | n.a. | Three MI modeling approaches were compared:  -jointly modeling the variables with missing values by sampling from the predictive distribution (here general location model as imputation model used)  -variable-by-variable approach (often used when joint modeling is not applicable)  -re-sampling based on an algorithm using bootstrap | A range of different analyses can be executed on the imputed datasets; generally good performance of all MI methods with minimal bias and good coverage, even in small samples and large variance for missing variable; joint modeling generally better efficiency but slightly more bias in small samples; re-sampling MI (non-parametric) outperforms parametric approaches in MNAR scenarios (but is still not good); under MAR sequential methods are better than joint modeling for small sample sizes | assumed data model (to  predict missing data) should be plausible and related to the analyst’s investigation; biased results under MNAR | Karakaya et al. 2015  The R package mice, mi and mix were used for multiple imputation. |
| Imputation-based Jackknife empirical likelihood (JEL) | MCAR | n.a. | 1. Hot deck imputation stratified by true disease status  2. estimate CIs for the ROC using jackknife empirical likelihood | Hot deck imputation maintains the distribution of the observed data; JEL has a similar performance as smoothed EL (SEL) regarding coverage probabilities; JEL simplifies the complexity of equations; narrow average length of CI | For hot deck (single) imputation in general: Underestimation of variability by using hot deck imputation; not adequate for MAR/MNAR | Yang & Zhao 2015  The authors state that the source code is available upon request. |
| Imputation-based profile-empirical likelihood (IPEL) | MCAR | n.a. | 1. hot deck imputation;  2. empirical likelihood to obtain CIs for the AUC based on imputed data | Hot deck imputation maintains the distribution of the observed data; consistent and asymptotically normal; simulation studies show:  good performance in moderate accuracy cases with even a small sample size | in high accuracy cases this method is conservative in small sample sizes and improves with increasing sample size; better in symmetric distributions than in asymmetric; see general comment above | Wang & Qin 2012  Programming code is not available. |
| Imputation-based profile empirical likelihood (IPEL) | MCAR | n.a. | 1. hot deck imputation;  2. log-empirical likelihood to obtain CIs for the sensitivity of ROC based on imputed data | hot deck imputation  retains distribution; performs well in moderate and large samples, even with a high missing rate; simple to implement; simulation studies: generally good performance | performance depends on  density estimates and may be inadequate in small samples, or those with high missingness probability; simulation studies: small coverage probabilities when small sample sizes and higher sensitivity; see general comment above | Wang & Qin 2014  Programming code is not available. |
| Imputation-based hybrid bootstrap empirical likelihood (IHBEL) | MCAR | n.a. | 1. hot deck imputation;  2. adapted bootstrap method on imputed data (executing random hot deck imputation procedure on bootstrap samples also)  3. empirical likelihood to obtain CIs for the sensitivity of ROC | appropriate for small  samples (better than IPEL); adapted bootstrap does not underestimate variance; performs well in moderate and large samples, even with a high missing rate; generally good performance | Performance decreases for low observation rate; see general comment above | Wang & Qin 2014  Programming code is not available. |
| Imputation-based smoothed semi-empirical likelihood approach | MCAR | n.a. | 1. random hot deck imputation for those with missing values in a test with non-parametric distribution (index or reference); MLE for a sample with a parametric distribution of test values (index or reference), then selecting random samples from the population with this estimated MLE parameter and choosing one random sample of this population  2. kernel smoothing technique to obtain semi-empirical likelihood-based CIs for the ROC | good coverage,  especially better than ignoring missing values in small sample sizes and large missingness probability; easy to calculate, and efficient to compute | see general comment above | Liu & Zhao 2012  Programming code is not available. |

^a^Strengths and Weaknesses are based on aspects which are mentioned by the authors of the respective method in their paper. In most cases, the proposed method has not been evaluated systematically by other studies. Hence, no other information on the performance of the respective method is available.

Table 7. Missing values in the **reference standard** and **multiple continuous index tests**

| **Method** | **Missingness Mechanism** | **CI/**  **CD** | **Description** | **Strengths^a^** | **Weaknesses^a^** | **Reference(s)** |
| --- | --- | --- | --- | --- | --- | --- |
| ROCKIT – comparing paired data with missing values | Unclear | unclear | Based on a parametric bivariate binormal model; ML estimation by the “method of scoring” applied; aims at comparing paired, partially paired and unpaired data  *Clin. app. (examples): Rosendahl et al. 2011; Vu et al. 2021* | ROCKIT can deal with both ordinal and continuous data; overall good performance, especially in “more realistic” cases where missing data is low; higher power when including unpaired data compared to excluding them from the analysis | In small samples or extreme cases, the model is liberal (alpha higher than 0.5); convergence problems when the number of diseased or non-diseased cases were really low or when index tests correlated highly | Metz et al. 1998  Link to software: [Users Guide for ROC-kit — Metz ROC Software (uchicago.edu)](http://metz-roc.uchicago.edu/MetzROC/software/users-guide) |

^a^Strengths and Weaknesses are based on aspects which are mentioned by the authors of the respective method in their paper. In most cases, the proposed method has not been evaluated systematically by other studies. Hence, no other information on the performance of the respective method is available.

1. **Inconclusive results in the index test**

Table 8. Inconclusive results in a **single categorical index test**

| **Method** | **Missing pattern** | **CI/**  **CD** | **Description** | **Strengths^a^** | **Weaknesses^a^** | **Reference(s)** |
| --- | --- | --- | --- | --- | --- | --- |
| Worst case scenario/ Intention to diagnose | Any pattern | n.a. | *Referring to Inconclusive results in general*  Inconclusive results are considered positive for non-diseased and negative for diseased patients  *Clin. App. (examples): Ooi et al. 2018; Staufer et al. 2019; Brodard et al. 2021; Li et al. 2021; de Vries et al. 2020; Wardziak et al. 2019* |  | Underestimates sensitivity and specificity; accuracy is substantially biased and extremely low coverage rates under MCAR and MAR (Ma et al. 2014) | Simel et al. 1987; Schuetz et al. 2012  No code or packages available / mentioned. |
| Positive/Negative imputation | Any pattern | n.a. | *Referring to Inconclusive results in general*  All Inconclusive results are considered either positive or negative  *Clin. App (examples): Subhas et al. 2012; Achenbach et al. 2005; Alkadhi et al. 2008; Garcia et al. 2006* |  | Positive imputation: overestimates sensitivity and underestimates specificity; negative imputation: reverse; accuracy is substantially biased and extremely low coverage rates under MCAR and MAR (Ma et al. 2014) | Schuetz et al. 2012; Shinkins et al. 2013  No code or packages available / mentioned. |
| 6 cell matrix  + conditional sensitivity/specificity | Any pattern | n.a. | *Referring to Inconclusive results in general or non-evaluable results*  6 cell matrix including inconclusive results in separate cells; conditional sensitivity and specificity are calculated by excluding inconclusive results from the analysis, but additionally, the test yield is given: the probability of obtaining either a positive or negative results of all test results (it can be further differentiated into YD+ (probability of a positive or negative result when the disease is present) and YD- (reverse)); LR+/- that gives the OR of an inconclusive result in diseased patients compared to those non-diseased patients; a decision on the clinical significance of inconclusive results can be assessed by calculating posterior odds = prior odds*(LR+/-) (Bayesian approach with a priori possible)  *Clin. app.: Jain et al. 2017; Crocker et al. 2020* | all results are presented; test yield can be estimated, and more realistic estimates of sensitivity & specificity | The additional parameters (test yield) may not be familiar to clinicians | Simel et al. 1987; 1991  No code or packages available / mentioned. |

^a^Strengths and Weaknesses are based on aspects which are mentioned by the authors of the respective method in their paper. In most cases, the proposed method has not been evaluated systematically by other studies. Hence, no other information on the performance of the respective method is available.

Table 9. Inconclusive results in a **single continuous index test**

| **Method** | **Missing pattern** | **CI/**  **CD** | **Description** | **Strengths^a^** | **Weaknesses^a^** | **Reference(s)** |
| --- | --- | --- | --- | --- | --- | --- |
| TG-ROC method (two graphs receiver operating characteristics curve) | unclear | n.a. | *Referring to Inconclusive results in general or more specific intermediate results*  Two cut-off values are defined by partitioning the index test values into positive, inconclusive, and negative based on the desired sensitivity and specificity values for the positive/negative values (excluding the inconclusive results); both cut-off values define an interval of inconclusive results that is excluded in the interpretation of test values and the calculation of sensitivity and specificity  *Clin. app. (examples): Druham et al. 2021; da Fonseca Junior et al. 2015; Jafarzadeh et al. 2004; Beck et al. 2005* | High accuracy for the valid outer ranges (excluding the inconclusive interval) | Lower power due to excluding those observations with values in the inconclusive interval; preselected desired values for sensitivity and specificity do often not match the final calculated parameters; change in prevalence estimate impacts PPV/ NPV; the valid ranges do not always maximize the number of correct decisions; inconclusive interval has sometimes ill-defined properties and shows not always a clear relationship with the area of overlap | Landsheer 2018; 2016; Greiner et al. 1995  Excel template by Greiner et al. 1995  Landsheer lists available software in their supplement: R package DiagnosisMed, MedCalc, SigmaPlot |
| Grey zone method | unclear | n.a. | *Referring to Inconclusive results in general or more specific intermediate results*  As in the TG-ROC method, an interval of inconclusive results (=grey zone) is defined that will be excluded in the interpretation and calculation of sensitivity and specificity; here, the two cut-off values are identified by defining desired positive and negative post-test probabilities (according to clinical implications); then Bayes theorem is used to calculate related likelihood ratios, sensitivity, specificity, and the needed cut-off values; CI for both cut-off values by bootstrap; if an interaction between index test and a covariate may be possible, then stratify by covariate before calculating the grey zone  *Clin. app (examples): Biais et al. 2014; Coste et al. 2006; Min et al. 2016; Bellanné-Chantelot et al. 2016; Giroti et al. 2007* | More restrictive than the TG-ROC method; gives high accuracy for the interval outside the grey zone; can be extended to ≥2 index tests: if testing sequentially the post-test probability of the first test replaces the pre-test probability of the second test; if testing simultaneously then a multidimensional graphical display of the grey zones can be constructed; no specific assumption/ distribution needed for only calculating the grey zone | Dependent on variance differences; lower power (observations excluded but less than in the TG-ROC method); difficult to obtain appropriate  values of LR, since it involves analysis of the screening context (to obtain pre-test probabilities, which vary given the context) and requirements for post-tests probabilities | Landsheer 2018; Coste & Pouchot 2003; Coste et al. 2006  SAS code for the grey zone by Landsheer 2018 (in the supplemental material), R function for calculating sensitivity, specificity and posttest probabilities is included in the R package UncertainInterval |
| Uncertain interval method | unclear | n.a. | *Referring to Inconclusive results in general*  Non-parametric approach; an interval of uncertainty is defined around the intersection of the distribution of index test values of the diseased and non-diseased; this intersection marks the point of highest uncertainty (this index test value cannot discriminate between diseased and non-diseased; values have a near equal probability of defining disease or non-disease); an interval around this intersection is defined by choosing sensitivity and specificity for this range (commonly 0.55, if higher than interval becomes larger); value range outside the uncertain interval is called more certain interval  *Clin. app.: Landsheer 2020; Stojadinovic et al. 2020* | Much less restrictive than the other both methods (TG-ROC, grey zone); more balanced regarding  the proportion of non-diseased and diseased patients that are excluded; the highest proportion of correctly diagnosed patients overall (compared to the TG-ROC and uncertain interval method), and the highest sample size (compared to the other methods); misclassifications are avoided and the method shows a reduced number of false decisions; there is a direct relationship between the strength of a test and the size of the interval | Lower accuracy than in the other two methods; a very strong test may prevent the determination of an uncertain interval; a weak test may lead to an exclusion of many observations which lie in the uncertain interval; the sample must be large enough (>200); unclear how the method performs in bi-normal distributions | Landsheer et al. 2016;2018; (Shinkins et al. 2013)  R Package by Landsheer: UncertainInterval (but removed from the repository: <https://cran.r-project.org/web/packages/UncertainInterval/index.html> )  R code as well as the R function uncertain.interval are available from Landsheer et al. 2016. |

^a^Strengths and Weaknesses are based on aspects which are mentioned by the authors of the respective method in their paper. In most cases, the proposed method has not been evaluated systematically by other studies. Hence, no other information on the performance of the respective method is available.

Table 10. Inconclusive results in **multiple categorical index tests**

| **Method** | **Missing pattern** | **CI/**  **CD** | **Description** | **Strengths^a^** | **Weaknesses^a^** | **Reference(s)** |
| --- | --- | --- | --- | --- | --- | --- |
| Bayesian random effects model for the intention-to-diagnose approach  (for meta-analyses) | unclear | unclear | *Referring to unevaluable results (in the sense of uninterpretable)*  inconclusive results were treated as a middle category in the presentation of results (constructing a 3x2 table) and analyzed according to the intention-to-diagnose principle (considered false positive and false negative for the non-diseased and diseased patients, respectively); in addition to common accuracy parameters, test yield is calculated as proposed by Simel et al. (1987); multivariate normal prior with large variances was used for the logit-transformed pooled probabilities, the chosen prior is uniform/ uninformative; for the random effects a multivariate normal prior was used |  | Sensitivity analyses should be conducted to investigate the influence of prior assumptions | Menke & Kowalski 2016  Source code not available. The command proc MCMC was used in SAS for the Bayesian analysis. |
| Extended TGLMM  (for meta-analyses) | MAR | CI | *Referring to unevaluable results* extended trivariate generalized linear mixed model (TGLMM) to account for missing data; sensitivity and specificity are considered dependent on disease prevalence; TGLMM models sensitivity and specificity together with prevalence; bias in PPV and NPV is avoided by adjusting for potential bias in disease prevalence; model includes a missingness probability (given true disease) parameter | TGLMM model and an approach that simply excludes missing values under MCAR give nearly unbiased results & good coverage (slightly lower for excluding); TGLMM produces better results under MAR than if missing values are simply excluded | TLGMM produces biased  results when univariate random effects are misspecified (Nikoloulopoulos 2020); conditional independence assumption | Ma et al. 2014;  SAS code for the TGLMM model (using the command proc nlmixed) is provided in the appendix. |
| trivariate vine copula mixed model  (for meta-analyses) | MAR | CI | *Referring to unevaluable results (in the sense of uninterpretable)*  This model uses a vine copula representation for the random effects distribution of the latent sensitivity, specificity, and prevalence; it uses bivariate parametric copulas with different tail dependence behavior; calculation of model parameters is done by ML | this model includes the  extended TGLMM as a special case; sensitivity, specificity & prevalence can be modeled in the original scale; tail dependencies and asymmetries can be provided; ML with true vine copula is highly efficient; MLE & SD rather robust to bivariate copula misspecification | MLE and SD are not robust to margin misspecification; conditional independence assumption; bivariate and trivariate copula mixed model which disregards non-evaluable results are biased and inefficient when the true model is the quadrivariate multinomial vine copula mixed model | Nikoloulopoulos 2020  The package CopulaREMADA is used; the code is included as an example in this package.<https://cran.r-project.org/web/packages/CopulaREMADA/CopulaREMADA.pdf> |
| multinomial quadrivariate D-vine copula mixed model  (for meta-analyses) | MAR | CI | *Referring to unevaluable results (in the sense of uninterpretable)*  The model includes the number of non-evaluable results as a separate outcome; regular vine copula for random effects are used; parameters are estimated by ML | The model uses all available data including non-evaluable results by including them as an outcome; ML is highly efficient; a low bias of results; ML estimates and SD are robust under copula misspecification | MLE and SD are not robust to margin misspecification; conditional independence assumption | Nikoloulopoulos 2020  The package CopulaREMADA is used; the code is included as an example in this package.<https://cran.r-project.org/web/packages/CopulaREMADA/CopulaREMADA.pdf> |

^a^Strengths and Weaknesses are based on aspects which are mentioned by the authors of the respective method in their paper. In most cases, the proposed method has not been evaluated systematically by other studies. Hence, no other information on the performance of the respective method is available.

Table 11. Inconclusive results in **multiple categorical index tests** and **an imperfect reference test**

| **Method** | **Missing pattern** | **CI/**  **CD** | **Description** | **Strengths^a^** | **Weaknesses^a^** | **Reference(s)** |
| --- | --- | --- | --- | --- | --- | --- |
| Traditional Latent Class Models (TLC) | unclear | CI | *Referring to intermediate results*  LCM to address an imperfect reference standard; inconclusive results must either be omitted or considered positive/ negative |  | performance depends on the handling of intermediate results: overestimated accuracy when omitted; substantial lower accuracy when considered positive/ negative; SEs cannot be calculated as there are too few degrees of freedom; produces biased results when conditional dependence present | Xu et al. 2013  URL link to source code is not valid anymore. |
| Extended log-linear LCM (LLLC) | unclear | CD | *Referring to intermediate results*  The model includes linear-by-linear interactions; in the case of 3 index tests, this leads to 4 additional parameters per test as each test has 3 different responses (positive, negative, and inconclusive); model parameters are calculated by ML | The calculation is straightforward; performance  similar to PLC (below) & less biased than the TLC model; it can incorporate more complicated dependence structures; better fit to the data then the TCM (indicates log-likelihood, correlation residuals close to 0) | interpretation is difficult; the addition of 4  parameters through interaction (might lead to non-identifiability); if all tests have intermediate results, then 3 tests are needed to achieve enough degrees of freedom; results are still slightly biased; the model is less efficient than the PLC model; sensitivity analyses are needed to explore different dependence structures | Xu et al. 2013  URL link to source code is not valid anymore. |
| Probit LCM (PLC) | unclear | CD | *Referring to intermediate results*  Assumes latent scale which is low for negative and high for positive test results, inconclusive results lie in the middle; two cut-off values are defined to differentiate between the three classes; conditional dependence is considered by using correlated latent quantities that are normally distributed; one constraint is set to the variance-covariance matrix to be a correlation matrix; ML estimation is inefficient and, therefore, an adapted expectation maximization algorithm (MCEM algorithm with Gibbs sampler) is used; parametric bootstrapping for SE calculation | interpretation is easier than for the LLLC; performance similar to LLLC model & less biased than the TLC model; parameter estimates are more efficient than those of the LLLC model (smaller variability); better fit to the data then the TCM (indicates log-likelihood, correlation residuals close to 0) | The calculation is difficult; the model per se is non-identifiable; therefore, constraints are necessary; it is assumed that observed test results are multivariate normal distributed; sensitivity analyses are needed to explore different dependence structures | Xu et al. 2013  URL link to source code is not valid anymore. |

^a^Strengths and Weaknesses are based on aspects which are mentioned by the authors of the respective method in their paper. In most cases, the proposed method has not been evaluated systematically by other studies. Hence, no other information on the performance of the respective method is available.

Table 12. Inconclusive results in **one categorical index test** and missing values in **the reference standard**

| **Method** | **Missing pattern** | **CI/**  **CD** | **Description** | **Strengths^a^** | **Weaknesses^a^** | **Reference(s)** |
| --- | --- | --- | --- | --- | --- | --- |
| Combined method: Simel et al. (1987) approach + Begg and Greene’s approach  Also applied: MI for handling inconclusive results | MAR for unverified patients  MAR for inconclusive results | n.a. | *Referring to indeterminate results and using differential verification*  Several analysis strategies are compared;  Considering only inconclusive results and ignoring differential verification (pooling results from gold standard and imperfect reference test):  -intention-to-diagnose for inconclusive results  -“best case”* for inconclusive results  -MI for inconclusive results using MICE and including available covariates  -Simel et al. (1987) approach (conditional sensitivity/specificity and test yield and LR for inconclusive results)  *Considering both inconclusive results and differential verification:*  -Simel et al. (1987) approach and Begg and Greene’s method for partial verification (results of the imperfect reference standard are considered as missing values)  *best-case: inconclusive results were considered positive for the diseased patients and negative for the non-diseased patients | *Focus on the combined method*  The combined method (Calculating conditional sensitivity/specificity + Begg and Greene’s method) yields the same results for accuracy parameters as using the Begg and Greene’s method only and excluding inconclusive results; furthermore, test yield and LR for inconclusive results were obtained and were similar to the Simel et al. approach only (which ignored the differential verification)  *MI for handling inconclusive results*  Values of accuracy parameters obtained through MI lay in between those obtained by the best case and intention-to-diagnose approach; the sensitivity calculated by the MI approach was slightly higher than in the combined approach; the specificity was similar | *Focus on the combined method*  As strategies were only compared on a clinical example and no simulation study was performed, the performance of the proposed strategy is difficult to assess | El Chamieh et al. 2022  Source code is not available. |

^a^Strengths and Weaknesses are based on aspects which are mentioned by the authors of the respective method in their paper. In most cases, the proposed method has not been evaluated systematically by other studies. Hence, no other information on the performance of the respective method is available.

Table 13. Other approaches to handling inconclusive results

| **Method** | **Missing pattern** | **CI/**  **CD** | **Description** | **Strengths^a^** | **Weaknesses^a^** | **Reference(s)** |
| --- | --- | --- | --- | --- | --- | --- |
| Inconclusive results in categorical index and reference standard:  3x3 table | Any pattern | n.a. | *Referring to Inconclusive results in general*  9 cell matrix with separate cells for inconclusive results in the index test and reference standard | Transparency in the presentation | No recommendation regarding the analysis | Schuetz et al. 2012  Programming code not necessary. |
| Inconclusive results (valid & invalid) in a categorical index test: clinical practice oriented | Any pattern | n.a. | *Referring to Inconclusive results in general but differentiating between invalid and valid inconclusive results*  Display of a 6-cell matrix including only valid inconclusive results and analyzing this table as inconclusive results are considered in practice; invalid inconclusive results (uninterpretable) are displayed separately by true disease status and are also analyzed as considered in practice | Transparency in the presentation | No clear recommendations for the analysis | Shinkins et al. 2013  Programming code not necessary. |
| Extended ROC | MCAR | n.a. | *Referring to uninterpretable results specifically*  Include inconclusive results (here uninterpretable) assuming they have a probability=0.5 to discriminate between disease and non-disease |  |  | Lu et al. 2002  Source code is not available. |

^a^Strengths and Weaknesses are based on aspects which are mentioned by the authors of the respective method in their paper. In most cases, the proposed method has not been evaluated systematically by other studies. Hence, no other information on the performance of the respective method is available.

1. **Full list of references included in this scoping review**

Albert, P. S. (2009). Estimating diagnostic accuracy of multiple binary tests with an imperfect reference standard. *Stat Med*, *28*(5), 780-797. https://doi.org/10.1002/sim.3514

Alonzo, T. A., & Pepe, M. S. (1999). Using a combination of reference tests to assess the accuracy of a new diagnostic test. *Stat Med*, *18*(22), 2987-3003. https://doi.org/10.1002/(sici)1097-0258(19991130)18:22<2987::aid-sim205>3.0.co;2-b

An, Y. (2012). Smoothed Empirical Likelihood Inference for ROC Curves with Missing Data. *Open Journal of Statistics*, *Vol.02No.01*, 7, Article 16880. https://doi.org/10.4236/ojs.2012.21003

Arifin, W. N., & Yusof, U. K. (2022). Correcting for partial verification bias in diagnostic accuracy studies: A tutorial using R. *Stat Med*, *41*(9), 1709-1727. https://doi.org/10.1002/sim.9311

Asselineau, J., Paye, A., Bessède, E., Perez, P., & Proust-Lima, C. (2018). Different latent class models were used and evaluated for assessing the accuracy of campylobacter diagnostic tests: overcoming imperfect reference standards? *Epidemiol Infect*, *146*(12), 1556-1564. https://doi.org/10.1017/s0950268818001723

Baker, S. G. (1995). Evaluating multiple diagnostic tests with partial verification. *Biometrics*, *51*(1), 330-337.

Bianco, A. M., Boente, G., González-Manteiga, W., & Pérez-González, A. (2023). Estimators for ROC curves with missing biomarkers values and informative covariates. *Stat Methods Appl.*

Boelaert, M., Aoun, K., Liinev, J., Goetghebeur, E., & Van der Stuyft, P. (1999). The potential of latent class analysis in diagnostic test validation for canine Leishmania infantum infection. *Epidemiol Infect*, *123*(3), 499-506. https://doi.org/10.1017/s0950268899003040

Buzoianu, M., & Kadane, J. B. (2008). Adjusting for verification bias in diagnostic test evaluation: a Bayesian approach. *Stat Med*, *27*(13), 2453-2473. https://doi.org/10.1002/sim.3099

Campbell, G., Pennello, G., & Yue, L. (2011). Missing data in the regulation of medical devices. *J Biopharm Stat*, *21*(2), 180-195. https://doi.org/10.1080/10543406.2011.550094

Cerullo, E., Jones, H. E., Carter, O., Quinn, T. J., Cooper, N. J., & Sutton, A. J. (2022). Meta-analysis of dichotomous and ordinal tests with an imperfect gold standard. *Research Synthesis Methods*, *n/a*(n/a). https://doi.org/https://doi.org/10.1002/jrsm.1567

Chen, S., Watson, P., & Parmigiani, G. (2005). Accuracy of MSI testing in predicting germline mutations of MSH2 and MLH1: a case study in Bayesian meta-analysis of diagnostic tests without a gold standard. *Biostatistics*, *6*(3), 450-464. https://doi.org/10.1093/biostatistics/kxi021

Cheng, W., & Tang, N. (2020). Smoothed empirical likelihood inference for ROC curve in the presence of missing biomarker values. *Biom J*, *62*(4), 1038-1059. https://doi.org/10.1002/bimj.201900121

Chi, Y.-Y., & Zhou, X.-H. (2008). Receiver operating characteristic surfaces in the presence of verification bias. *Journal of the Royal Statistical Society Series C-Applied Statistics*, *57*, 1-23. https://doi.org/10.1111/j.1467-9876.2007.00597.x

Chikere, C. M. U., Wilson, K., Graziadio, S., Vale, L., & Allen, A. J. (2019). Diagnostic test evaluation methodology: A systematic review of methods employed to evaluate diagnostic tests in the absence of gold standard - An update. *PLoS One*, *14*(10), e0223832. https://doi.org/10.1371/journal.pone.0223832

Chikere, C. M. U., Wilson, K. J., Allen, A. J., & Vale, L. (2021). Comparative diagnostic accuracy studies with an imperfect reference standard - a comparison of correction methods. *BMC Med Res Methodol*, *21*(1), 67. https://doi.org/10.1186/s12874-021-01255-4

Cho, H., Matthews, G. J., & Harel, O. (2019). Confidence Intervals for the Area Under the Receiver Operating Characteristic Curve in the Presence of Ignorable Missing Data. *Int Stat Rev*, *87*(1), 152-177. https://doi.org/10.1111/insr.12277

Cipriani, D., Fox, C., Khuder, S., & Boudreau, N. (2005). Comparing Rasch analyses probability estimates to sensitivity, specificity and likelihood ratios when examining the utility of medical diagnostic tests. *J Appl Meas*, *6*(2), 180-201. https://digitalcommons.chapman.edu/cgi/viewcontent.cgi?article=1038&context=pt_articles

Coart, E., Barrado, L. G., Duits, F. H., Scheltens, P., van der Flier, W. M., Teunissen, C. E., van der Vies, S. M., & Burzykowski, T. (2015). Correcting for the Absence of a Gold Standard Improves Diagnostic Accuracy of Biomarkers in Alzheimer's Disease. *J Alzheimers Dis*, *46*(4), 889-899. https://doi.org/10.3233/jad-142886

Collins, J., & Albert, P. S. (2016). Estimating diagnostic accuracy without a gold standard: A continued controversy. *J Biopharm Stat*, *26*(6), 1078-1082. https://doi.org/10.1080/10543406.2016.1226334

Collins, J., & Huynh, M. (2014). Estimation of diagnostic test accuracy without full verification: a review of latent class methods. *Stat Med*, *33*(24), 4141-4169. https://doi.org/10.1002/sim.6218

Coste, J., Jourdain, P., & Pouchot, J. (2006). A gray zone assigned to inconclusive results of quantitative diagnostic tests: Application to the use of brain natriuretic peptide for diagnosis of heart failure in acute dyspneic patients. *Clin Chem*, *52*(12), 2229-2235. https://doi.org/10.1373/clinchem.2006.072280

Coste, J., & Pouchot, J. (2003). A grey zone for quantitative diagnostic and screening tests. *Int J Epidemiol*, *32*(2), 304-313. https://doi.org/10.1093/ije/dyg054

de Groot, J. A., Dendukuri, N., Janssen, K. J., Reitsma, J. B., Bossuyt, P. M., & Moons, K. G. (2011). Adjusting for differential-verification bias in diagnostic-accuracy studies: a Bayesian approach. *Epidemiology*, *22*(2), 234-241. https://doi.org/10.1097/EDE.0b013e318207fc5c

de Groot, J. A., Dendukuri, N., Janssen, K. J., Reitsma, J. B., Brophy, J., Joseph, L., Bossuyt, P. M., & Moons, K. G. (2012). Adjusting for partial verification or workup bias in meta-analyses of diagnostic accuracy studies. *Am J Epidemiol*, *175*(8), 847-853. https://doi.org/10.1093/aje/kwr383

de Groot, J. A., Janssen, K. J., Zwinderman, A. H., Bossuyt, P. M., Reitsma, J. B., & Moons, K. G. (2011). Correcting for partial verification bias: a comparison of methods. *Ann Epidemiol*, *21*(2), 139-148. https://doi.org/10.1016/j.annepidem.2010.10.004

de Groot, J. A., Janssen, K. J., Zwinderman, A. H., Moons, K. G., & Reitsma, J. B. (2008). Multiple imputation to correct for partial verification bias revisited. *Stat Med*, *27*(28), 5880-5889. https://doi.org/10.1002/sim.3410

El Chamieh, C., Vielh, P., & Chevret, S. (2022). Statistical methods for evaluating the fine needle aspiration cytology procedure in breast cancer diagnosis. *BMC Med Res Methodol*, *22*(1), 40. https://doi.org/10.1186/s12874-022-01506-y

Engel, B., Swildens, B., Stegeman, A., Buist, W., & de Jong, M. (2006). Estimation of sensitivity and specificity of three conditionally dependent diagnostic tests in the absence of a gold standard. *Journal of Agricultural Biological and Environmental Statistics*, *11*(4), 360-380. https://doi.org/10.1198/108571106x153534

Enøe, C., Georgiadis, M. P., & Johnson, W. O. (2000). Estimation of sensitivity and specificity of diagnostic tests and disease prevalence when the true disease state is unknown. *Prev Vet Med*, *45*(1-2), 61-81. https://doi.org/10.1016/s0167-5877(00)00117-3

Gad, A. M., Ali, A. A. M., & Mohamed, R. H. (2022). A multiple imputation approach to evaluate the accuracy of diagnostic tests in presence of missing values. *Communications in Mathematical Biology and Neuroscience*.

García Barrado, L., Coart, E., & Burzykowski, T. (2016). Development of a diagnostic test based on multiple continuous biomarkers with an imperfect reference test. *Stat Med*, *35*(4), 595-608. https://doi.org/10.1002/sim.6733

García Barrado, L., Coart, E., & Burzykowski, T. (2017). Estimation of diagnostic accuracy of a combination of continuous biomarkers allowing for conditional dependence between the biomarkers and the imperfect reference-test. *Biometrics*, *73*(2), 646-655. https://doi.org/10.1111/biom.12583

Ghaemmaghami, P., Ayatollahi, S. M. T., Bagheri, Z., & Jafarzadeh, S. R. (2021). Covariate-adjusted Bayesian estimation of the performance of a continuous diagnostic test with a limit of detection in the absence of a reference standard: a simulation study. *Communications in Statistics-Simulation and Computation*. https://doi.org/10.1080/03610918.2021.1881117

Greiner, M. (1995). Two-graph receiver operating characteristic (TG-ROC): a Microsoft-EXCEL template for the selection of cut-off values in diagnostic tests. *J Immunol Methods*, *185*(1), 145-146. https://doi.org/10.1016/0022-1759(95)00078-o

Greiner, M., Sohr, D., & Göbel, P. (1995). A modified ROC analysis for the selection of cut-off values and the definition of intermediate results of serodiagnostic tests. *Journal of Immunological Methods*, *185*(1), 123-132. https://doi.org/https://doi.org/10.1016/0022-1759(95)00121-P

Hahn, A., Schwarz, N. G., & Frickmann, H. (2019). Comparison of screening tests without a gold standard-A pragmatic approach with virtual reference testing. *Acta Trop*, *199*, 105118. https://doi.org/10.1016/j.actatropica.2019.105118

Hajivandi, A., Shirazi, H. R. G., Saadat, S. H., & Chehrazi, M. (2018). A Bayesian Analysis With Informative Prior on Disease Prevalence for Predicting Missing Values Due To Verification Bias. *Open Access Maced J Med Sci*, *6*(7), 1225-1230. https://doi.org/10.3889/oamjms.2018.296

He, H., & McDermott, M. P. (2012). A robust method using propensity score stratification for correcting verification bias for binary tests. *Biostatistics*, *13*(1), 32-47. https://doi.org/10.1093/biostatistics/kxr020

Held, U., Brunner, F., Steurer, J., & Wertli, M. M. (2015). Bayesian meta-analysis of test accuracy in the absence of a perfect reference test applied to bone scintigraphy for the diagnosis of complex regional pain syndrome. *Biom J*, *57*(6), 1020-1037. https://doi.org/10.1002/bimj.201400155

Helman, S. K., Mummah, R. O., Gostic, K. M., Buhnerkempe, M. G., Prager, K. C., & Lloyd-Smith, J. O. (2020). Estimating prevalence and test accuracy in disease ecology: How Bayesian latent class analysis can boost or bias imperfect test results. *Ecology and Evolution*, *10*(14), 7221-7232. https://doi.org/10.1002/ece3.6448

Jafarzadeh, S. R., Johnson, W. O., & Gardner, I. A. (2016). Bayesian modeling and inference for diagnostic accuracy and probability of disease based on multiple diagnostic biomarkers with and without a perfect reference standard. *Stat Med*, *35*(6), 859-876. https://doi.org/10.1002/sim.6745

Johnson, W. O., Jones, G., & Gardner, I. A. (2019). Gold standards are out and Bayes is in: Implementing the cure for imperfect reference tests in diagnostic accuracy studies. *Prev Vet Med*, *167*, 113-127. https://doi.org/10.1016/j.prevetmed.2019.01.010

Karakaya, J., Karabulut, E., & Yucel, R. M. (2015). Sensitivity to imputation models and assumptions in receiver operating characteristic analysis with incomplete data. *J Stat Comput Simul*, *85*(17), 3498-3511. https://doi.org/10.1080/00949655.2014.983111

Kosinski, A. S., & Barnhart, H. X. (2003). A global sensitivity analysis of performance of a medical diagnostic test when verification bias is present. *Stat Med*, *22*(17), 2711-2721. https://doi.org/10.1002/sim.1517

Landsheer, J. A. (2016). Interval of Uncertainty: An Alternative Approach for the Determination of Decision Thresholds, with an Illustrative Application for the Prediction of Prostate Cancer. *PLoS One*, *11*(11), e0166007. https://doi.org/10.1371/journal.pone.0166007

Landsheer, J. A. (2018). The Clinical Relevance of Methods for Handling Inconclusive Medical Test Results: Quantification of Uncertainty in Medical Decision-Making and Screening. *Diagnostics (Basel)*, *8*(2). https://doi.org/10.3390/diagnostics8020032

Li, H., & Gatsonis, C. (2019). Combining biomarker trajectories to improve diagnostic accuracy in prospective cohort studies with verification bias. *Stat Med*, *38*(11), 1968-1990. https://doi.org/10.1002/sim.8079

Li, S., & Ning, Y. (2015). Estimation of covariate-specific time-dependent ROC curves in the presence of missing biomarkers. *Biometrics*, *71*(3), 666-676. https://doi.org/10.1111/biom.12312

Lian, Q., Hodges, J. S., & Chu, H. (2019). A Bayesian Hierarchical Summary Receiver Operating Characteristic Model for Network Meta-analysis of Diagnostic Tests. *J Am Stat Assoc*, *114*(527), 949-961. https://doi.org/10.1080/01621459.2018.1476239

Lin, C. Y., Barnhart, H. X., & Kosinski, A. S. (2006). The weighted generalized estimating equations approach for the evaluation of medical diagnostic test at subunit level. *Biom J*, *48*(5), 758-771. https://doi.org/10.1002/bimj.200510199

Lin, R., Chan, K. G., & Shi, H. (2021). A unified Bayesian framework for exact inference of area under the receiver operating characteristic curve. *Stat Methods Med Res*, *30*(10), 2269-2287. https://doi.org/10.1177/09622802211037070

Liu, D., & Zhou, X. H. (2011). Semiparametric estimation of the covariate-specific ROC curve in presence of ignorable verification bias. *Biometrics*, *67*(3), 906-916. https://doi.org/10.1111/j.1541-0420.2011.01562.x

Liu, X., & Zhao, Y. (2012). Semi-empirical likelihood inference for the ROC curve with missing data. *Journal of Statistical Planning and Inference*, *142*(12), 3123-3133. https://doi.org/10.1016/j.jspi.2012.06.011

Liu, Y., Chen, Y., & Chu, H. (2015). A unification of models for meta-analysis of diagnostic accuracy studies without a gold standard. *Biometrics*, *71*(2), 538-547. https://doi.org/10.1111/biom.12264

Liu, Y. L., Ying, G. S., Quinn, G. E., Zhou, X. H., & Chen, Y. (2022). Extending Hui-Walter framework to correlated outcomes with application to diagnosis tests of an eye disease among premature infants. *Stat Med*, *41*(3), 433-448. https://doi.org/10.1002/sim.9269

Long, Q., Zhang, X., & Hsu, C.-H. (2011b). Nonparametric multiple imputation for receiver operating characteristics analysis when some biomarker values are missing at random. *Statistics in medicine*, *30*(26), 3149-3161. https://doi.org/https://doi.org/10.1002/sim.4338

Long, Q., Zhang, X., & Johnson, B. A. (2011a). Robust estimation of area under ROC curve using auxiliary variables in the presence of missing biomarker values. *Biometrics*, *67*(2), 559-567. https://doi.org/10.1111/j.1541-0420.2010.01487.x

Lu, Y., Heller, D. N., & Zhao, S. (2002). Receiver operating characteristic (ROC) analysis for diagnostic examinations with uninterpretable cases. *Stat Med*, *21*(13), 1849-1865. https://doi.org/10.1002/sim.1143

Ma, X., Chen, Y., Cole, S. R., & Chu, H. (2016). A hybrid Bayesian hierarchical model combining cohort and case-control studies for meta-analysis of diagnostic tests: Accounting for partial verification bias. *Stat Methods Med Res*, *25*(6), 3015-3037. https://doi.org/10.1177/0962280214536703

Ma, X., K Suri, M. F., & Chu, H. (2014). A trivariate meta-analysis of diagnostic studies accounting for prevalence and non-evaluable subjects: re-evaluation of the meta-analysis of coronary CT angiography studies. *BMC medical research methodology*, *14*(1), 128. https://doi.org/10.1186/1471-2288-14-128

Ma, X., Lian, Q., Chu, H., Ibrahim, J. G., & Chen, Y. (2018). A Bayesian hierarchical model for network meta-analysis of multiple diagnostic tests. *Biostatistics*, *19*(1), 87-102. https://doi.org/10.1093/biostatistics/kxx025

Marin-Jimenez, A. E., & Roldan-Nofuentes, J. A. (2014). Global hypothesis test to compare the likelihood ratios of multiple binary diagnostic tests with ignorable missing data. *Sort-Statistics and Operations Research Transactions*, *38*(2), 305-323. <Go to ISI>://WOS:000346689100011

Marin-Jimenez, A. E., & Roldan-Nofuentes, J. A. (2017). COMPARISON OF THE PREDICTIVE VALUES OF MULTIPLE BINARY DIAGNOSTIC TESTS IN THE PRESENCE OF IGNORABLE MISSING DATA. *Revstat-Statistical Journal*, *15*(1), 45-64. <Go to ISI>://WOS:000398033200003

Martinez-Camblor, P. (2013). Area under the ROC curve comparison in the presence of missing data. *Journal of the Korean Statistical Society*, *42*(4), 431-442. https://doi.org/10.1016/j.jkss.2013.01.004

Mattner, L., & Mattner, F. (2013). Confidence bounds for the sensitivity lack of a less specific diagnostic test, without gold standard. *Metrika*, *76*(2), 239-263. https://doi.org/10.1007/s00184-012-0385-9

McDonald, J. L., & Hodgson, D. J. (2018). Prior Precision, Prior Accuracy, and the Estimation of Disease Prevalence Using Imperfect Diagnostic Tests. *Frontiers in Veterinary Science*, *5*, Article 83. https://doi.org/10.3389/fvets.2018.00083

Menke, J., & Kowalski, J. (2016). Diagnostic accuracy and utility of coronary CT angiography with consideration of unevaluable results: A systematic review and multivariate Bayesian random-effects meta-analysis with intention to diagnose. *European Radiology*, *26*(2), 451-458. https://doi.org/10.1007/s00330-015-3831-z

Menten, J., Boelaert, M., & Lesaffre, E. (2008). Bayesian latent class models with conditionally dependent diagnostic tests: a case study. *Stat Med*, *27*(22), 4469-4488. https://doi.org/10.1002/sim.3317

Menten, J., & Lesaffre, E. (2015). A general framework for comparative Bayesian meta-analysis of diagnostic studies. *BMC Med Res Methodol*, *15*, 70. https://doi.org/10.1186/s12874-015-0061-7

Metz, C. E., Herman, B. A., & Roe, C. A. (1998). Statistical comparison of two ROC-curve estimates obtained from partially-paired datasets. *Med Decis Making*, *18*(1), 110-121. https://doi.org/10.1177/0272989x9801800118

Montero-Alonso, M. A., & Roldán-Nofuentes, J. A. (2019). Approximate confidence intervals for the likelihood ratios of a binary diagnostic test in the presence of partial disease verification. *J Biopharm Stat*, *29*(1), 56-81. https://doi.org/10.1080/10543406.2018.1452025

Naaktgeboren, C. A., de Groot, J. A., Rutjes, A. W., Bossuyt, P. M., Reitsma, J. B., & Moons, K. G. (2016). Anticipating missing reference standard data when planning diagnostic accuracy studies. *Bmj*, *352*, i402. https://doi.org/10.1136/bmj.i402

Nikoloulopoulos, A. K. (2020a). An extended trivariate vine copula mixed model for meta-analysis of diagnostic studies in the presence of non-evaluable outcomes. *The International Journal of Biostatistics*, *16*(2). https://doi.org/doi:10.1515/ijb-2019-0107

Nikoloulopoulos, A. K. (2020b). A multinomial quadrivariate D-vine copula mixed model for meta-analysis of diagnostic studies in the presence of non-evaluable subjects. *Statistical methods in medical research*, *29*(10), 2988-3005. https://doi.org/10.1177/0962280220913898

Paulino, C. D., & Silva, G. L. (2019). Bayesian comparison of diagnostic tests with largely non-informative missing data. *Journal of Statistical Computation and Simulation*, *89*(10), 1877-1886. https://doi.org/10.1080/00949655.2019.1601726

Pence, B. W., Miller, W. C., & Gaynes, B. N. (2009). Prevalence estimation and validation of new instruments in psychiatric research: an application of latent class analysis and sensitivity analysis. *Psychol Assess*, *21*(2), 235-239. https://doi.org/10.1037/a0015686

Pennello, G. A. (2011). Bayesian analysis of diagnostic test accuracy when disease state is unverified for some subjects. *J Biopharm Stat*, *21*(5), 954-970. https://doi.org/10.1080/10543406.2011.590921

Poleto, F. Z., Singer, J. M., & Paulino, C. D. (2011). Comparing diagnostic tests with missing data. *Journal of Applied Statistics*, *38*(6), 1207-1222, Article Pii 935118198. https://doi.org/10.1080/02664763.2010.491860

Qu, Y. S., & Hadgu, A. (1998). A model for evaluating sensitivity and specificity for correlated diagnostic tests in efficacy studies with an imperfect reference test. *Journal of the American Statistical Association*, *93*(443), 920-928. https://doi.org/10.2307/2669830

Reitsma, J. B., Rutjes, A. W., Khan, K. S., Coomarasamy, A., & Bossuyt, P. M. (2009). A review of solutions for diagnostic accuracy studies with an imperfect or missing reference standard. *J Clin Epidemiol*, *62*(8), 797-806. https://doi.org/10.1016/j.jclinepi.2009.02.005

Roldan-Nofuentes, J. A. (2021). Computational methods to simultaneously compare the predictive values of two diagnostic tests with missing data: EM-SEM algorithms and multiple imputation. *Figshare*. https://doi.org/http://dx.doi.org/10.6084/m9.figshare.14602706.v1

Roldan-Nofuentes, J. A., & Luna del Castillo, J. D. (2005). Comparing the likelihood ratios of two binary diagnostic tests in the presence of partial verification. *Biom J*, *47*(4), 442-457. https://doi.org/10.1002/bimj.200410134

Roldan-Nofuentes, J. A., Luna del Castillo, J. D., & Femia Marzo, P. (2009). Computational methods for comparing two binary diagnostic tests in the presence of partial verification of the disease. *Computational Statistics*, *24*(4), 695-718. https://doi.org/10.1007/s00180-009-0155-y

Rotnitzky, A., Faraggi, D., & Schisterman, E. (2006). Doubly robust estimation of the area under the receiver-operating characteristic curve in the presence of verification bias. *Journal of the American Statistical Association*, *101*(475), 1276-1288. https://doi.org/10.1198/016214505000001339

Rutjes, A. W., Reitsma, J. B., Coomarasamy, A., Khan, K. S., & Bossuyt, P. M. (2007). Evaluation of diagnostic tests when there is no gold standard. A review of methods. *Health Technol Assess*, *11*(50), iii, ix-51. https://doi.org/10.3310/hta11500

Rutjes, A. W., Reitsma, J. B., Di Nisio, M., Smidt, N., van Rijn, J. C., & Bossuyt, P. M. (2006). Evidence of bias and variation in diagnostic accuracy studies. *Cmaj*, *174*(4), 469-476. https://doi.org/10.1503/cmaj.050090

Schneeweiss, S. (2000). Sensitivity analysis of the diagnostic value of endoscopies in cross-sectional studies in the absence of a gold standard. *Int J Technol Assess Health Care*, *16*(3), 834-841. https://doi.org/10.1017/s0266462300102107

Schuetz, G. M., Schlattmann, P., & Dewey, M. (2012). Use of 3×2 tables with an intention to diagnose approach to assess clinical performance of diagnostic tests: meta-analytical evaluation of coronary CT angiography studies. *BMJ : British Medical Journal*, *345*, e6717. https://doi.org/10.1136/bmj.e6717

Shinkins, B., Thompson, M., Mallett, S., & Perera, R. (2013). Diagnostic accuracy studies: how to report and analyse inconclusive test results. *Bmj*, *346*, f2778. https://doi.org/10.1136/bmj.f2778

Siadaty, M. S., Philbrick, J. T., Heim, S. W., & Schectman, J. M. (2004). Repeated-measures modeling improved comparison of diagnostic tests in meta-analysis of dependent studies. *J Clin Epidemiol*, *57*(7), 698-711. https://doi.org/10.1016/j.jclinepi.2003.12.007

Simel, D. L., Feussner, J. R., DeLong, E. R., & Matchar, D. B. (1987). Intermediate, indeterminate, and uninterpretable diagnostic test results. *Med Decis Making*, *7*(2), 107-114. https://doi.org/10.1177/0272989x8700700208

Simel, D. L., Matchar, D. B., & Feussner, J. R. (1991). Diagnostic tests are not always black or white: Or, all that glitters is not [A] gold [standard]. *Journal of Clinical Epidemiology*, *44*(9), 967-970. https://doi.org/https://doi.org/10.1016/0895-4356(91)90065-H

Tang, S., Hemyari, P., Canchola, J. A., & Duncan, J. (2018). Dual composite reference standards (dCRS) in molecular diagnostic research: A new approach to reduce bias in the presence of Imperfect reference. *J Biopharm Stat*, *28*(5), 951-965. https://doi.org/10.1080/10543406.2018.1428613

Unal, I., & Burgut, H. R. (2014). Verification bias on sensitivity and specificity measurements in diagnostic medicine: a comparison of some approaches used for correction. *Journal of Applied Statistics*, *41*(5), 1091-1104. https://doi.org/10.1080/02664763.2013.862217

van Geloven, N., Brooze, K. A., Opmeer, B. C., Mol, B. W., & Zwinderman, A. H. (2012). How to deal with double partial verification when evaluating two index tests in relation to a reference test? *Stat Med*, *31*(11-12), 1265-1276. https://doi.org/10.1002/sim.4440

van Smeden, M., Naaktgeboren, C. A., Reitsma, J. B., Moons, K. G., & de Groot, J. A. (2014). Latent class models in diagnostic studies when there is no reference standard--a systematic review. *Am J Epidemiol*, *179*(4), 423-431. https://doi.org/10.1093/aje/kwt286

Walsh, T. (2018). Fuzzy gold standards: Approaches to handling an imperfect reference standard. *J Dent*, *74 Suppl 1*, S47-s49. https://doi.org/10.1016/j.jdent.2018.04.022

Wang, B., & Qin, G. (2012). Imputation-based empirical likelihood inference for the area under the ROC curve with missing data. *Statistics and Its Interface*, *5*(3), 319-329. <Go to ISI>://WOS:000308969400005

Wang, B., & Qin, G. (2014). Empirical Likelihood-Based Confidence Intervals for the Sensitivity of a Continuous-Scale Diagnostic Test with Missing Data. *Communications in Statistics-Theory and Methods*, *43*(15), 3248-3268. https://doi.org/10.1080/03610926.2012.695849

Wang, C., Lin, X., & Nelson, K. P. (2020). Bayesian hierarchical latent class models for estimating diagnostic accuracy. *Stat Methods Med Res*, *29*(4), 1112-1128. https://doi.org/10.1177/0962280219852649

Wang, Y., Chen, H., Zeng, D., Mauro, C., Duan, N., & Shear, M. K. (2013). Auxiliary marker-assisted classification in the absence of class identifiers. *J Am Stat Assoc*, *108*(502), 553-565. https://doi.org/10.1080/01621459.2013.775949

Xu, H., Black, M. A., & Craig, B. A. (2013). Evaluating accuracy of diagnostic tests with intermediate results in the absence of a gold standard. *Stat Med*, *32*(15), 2571-2584. https://doi.org/10.1002/sim.5695

Yang, H., & Zhao, Y. (2015). Smoothed jackknife empirical likelihood inference for ROC curves with missing data. *Journal of Multivariate Analysis*, *140*, 123-138. https://doi.org/https://doi.org/10.1016/j.jmva.2015.05.002

Yu, Q., Tang, W., Ma, Y., Gamble, S. A., & Tu, X. M. (2008). Comparing Multiple Sensitivities and Specificities with Different Diagnostic Criteria: Applications to Sexual Abuse and Sexual Health Research. *Comput Stat Data Anal*, *53*(1), 27-37. https://doi.org/10.1016/j.csda.2008.05.031

Yu, W., Kim, J. K., & Park, T. (2018). Estimation of Area Under the ROC Curve under nonignorable verification bias. *Stat Sin*, *28*(4), 2149-2166. https://doi.org/10.5705/ss.202016.0315

Zhang, B., Chen, Z., & Albert, P. S. (2012). Estimating diagnostic accuracy of raters without a gold standard by exploiting a group of experts. *Biometrics*, *68*(4), 1294-1302. https://doi.org/10.1111/j.1541-0420.2012.01789.x

Zhang, Y. (2013). *Statistical methods for evaluating the diagnostic accuracy of incomplete multiple tests* (Publication Number 3594244) [Dr.P.H., The University of North Carolina at Chapel Hill]. ProQuest Dissertations & Theses Global. Ann Arbor. https://www.proquest.com/dissertations-theses/statistical-methods-evaluating-diagnostic/docview/1439912109/se-2?accountid=11262

https://sfx-49gbv.hosted.exlibrisgroup.com/sfx_subhh?url_ver=Z39.88-2004&rft_val_fmt=info:ofi/fmt:kev:mtx:dissertation&genre=dissertations&sid=ProQ:ProQuest+Dissertations+%26+Theses+Global&atitle=&title=Statistical+methods+for+evaluating+the+diagnostic+accuracy+of+incomplete+multiple+tests&issn=&date=2013-01-01&volume=&issue=&spage=&au=Zhang%2C+Yi&isbn=978-1-303-37935-2&jtitle=&btitle=&rft_id=info:eric/&rft_id=info:doi/

Zhang, Y., Chu, H., & Zeng, D. (2014). Evaluation of incomplete multiple diagnostic tests, with an application in the colon cancer family registry study. *Journal of Applied Statistics*, *41*(3), 688-700. https://doi.org/10.1080/02664763.2013.849231

Zhou, X. H., & Gatsonis, C. A. (1996). A simple method for comparing correlated ROC curves using incomplete data. *Stat Med*, *15*(15), 1687-1693. https://doi.org/10.1002/(sici)1097-0258(19960815)15:15<1687::Aid-sim324>3.0.Co;2-s

1. **References listed under clinical application**

Achenbach, S., Ropers, D., Pohle, F.-K., Raaz, D., von Erffa, J., Yilmaz, A., Muschiol, G., & Daniel, W. G. (2005). Detection of coronary artery stenoses using multi-detector CT with 16×0.75 collimation and 375 ms rotation. *European heart journal*, *26*(19), 1978-1986. https://doi.org/10.1093/eurheartj/ehi326

Alkadhi, H., Scheffel, H., Desbiolles, L., Gaemperli, O., Stolzmann, P., Plass, A., Goerres, G. W., Luescher, T. F., Genoni, M., Marincek, B., Kaufmann, P. A., & Leschka, S. (2008). Dual-source computed tomography coronary angiography: influence of obesity, calcium load, and heart rate on diagnostic accuracy. *European heart journal*, *29*(6), 766-776. https://doi.org/10.1093/eurheartj/ehn044

Beck, R., Gaspar, A., Mihaljević, Z., Marinculić, A., Stojcević, D., & Brstilo, M. (2005). Evaluation of ELISA for detection of Trichinella antibodies in muscle juice samples of naturally infected pigs. *Vet Parasitol*, *132*(1-2), 91-95. https://doi.org/10.1016/j.vetpar.2005.05.034

Bellanné-Chantelot, C., Coste, J., Ciangura, C., Fonfrède, M., Saint-Martin, C., Bouché, C., Sonnet, E., Valéro, R., Lévy, D. J., Dubois-Laforgue, D., & Timsit, J. (2016). High-sensitivity C-reactive protein does not improve the differential diagnosis of HNF1A-MODY and familial young-onset type 2 diabetes: A grey zone analysis. *Diabetes Metab*, *42*(1), 33-37. https://doi.org/10.1016/j.diabet.2015.02.001

Benedikt, R. A., Boatsman, J. E., Swann, C. A., Kirkpatrick, A. D., & Toledano, A. Y. (2017). Concurrent Computer-Aided Detection Improves Reading Time of Digital Breast Tomosynthesis and Maintains Interpretation Performance in a Multireader Multicase Study. *American Journal of Roentgenology*, *210*(3), 685-694. https://doi.org/10.2214/AJR.17.18185

Biais, M., Ehrmann, S., Mari, A., Conte, B., Mahjoub, Y., Desebbe, O., Pottecher, J., Lakhal, K., Benzekri-Lefevre, D., Molinari, N., Boulain, T., Lefrant, J.-Y., Muller, L., & with the collaboration of AzuRea, G. (2014). Clinical relevance of pulse pressure variations for predicting fluid responsiveness in mechanically ventilated intensive care unit patients: the grey zone approach. *Critical Care*, *18*(6), 587. https://doi.org/10.1186/s13054-014-0587-9

Brodard, J., Benites, V., Stalder Zeerleder, D., & Nagler, M. (2021). Accuracy of the functional, flow cytometer-based Emo-Test HIT Confirm® for the diagnosis of heparin-induced thrombocytopenia. *Thrombosis Research*, *203*, 22-26. https://doi.org/https://doi.org/10.1016/j.thromres.2021.04.017

Conant, E. F., Toledano, A. Y., Periaswamy, S., Fotin, S. V., Go, J., Boatsman, J. E., & Hoffmeister, J. W. (2019). Improving Accuracy and Efficiency with Concurrent Use of Artificial Intelligence for Digital Breast Tomosynthesis. *Radiol Artif Intell*, *1*(4), e180096. https://doi.org/10.1148/ryai.2019180096

Coste, J., Jourdain, P., & Pouchot, J. (2006). A gray zone assigned to inconclusive results of quantitative diagnostic tests: Application to the use of brain natriuretic peptide for diagnosis of heart failure in acute dyspneic patients. *Clin Chem*, *52*(12), 2229-2235. https://doi.org/10.1373/clinchem.2006.072280

Crocker, C., Akl, M., Abdolell, M., Kamali, M., & Costa, A. F. (2020). Ultrasound and CT in the Diagnosis of Appendicitis: Accuracy With Consideration of Indeterminate Examinations According to STARD Guidelines. *American Journal of Roentgenology*, *215*(3), 639-644. https://doi.org/10.2214/AJR.19.22370

da Fonseca Junior, J. H., Pitta, G. B., & Miranda Júnior, F. (2015). Accuracy of doppler ultrasonography in the evaluation of hemodialysis arteriovenous fistula maturity. *Rev Col Bras Cir*, *42*(3), 138-142. https://doi.org/10.1590/0100-69912015003002

de Vries, A. B., van der Heide, F., ter Steege, R. W. F., Koornstra, J. J., Buddingh, K. T., Gouw, A. S. H., & Weersma, R. K. (2020). Limited diagnostic accuracy and clinical impact of single-operator peroral cholangioscopy for indeterminate biliary strictures. *Endoscopy*, *52*(02), 107-114.

Durham, A. E., Clarke, B. R., Potier, J. F. N., Hammarstrand, R., & Malone, G. L. (2021). Clinically and temporally specific diagnostic thresholds for plasma ACTH in the horse. *Equine Veterinary Journal*, *53*(2), 250-260. https://doi.org/https://doi.org/10.1111/evj.13292

Garcia, M. J., Lessick, J., Hoffmann, M. H. K., & CATSCAN Study Investigators, f. t. (2006). Accuracy of 16-Row Multidetector Computed Tomography for the Assessment of Coronary Artery Stenosis. *JAMA*, *296*(4), 403-411. https://doi.org/10.1001/jama.296.4.403

Giroti, R. I., Verma, S., Singh, K., Malik, R., & Talwar, I. (2007). A grey zone approach for evaluation of 15 short tandem repeat loci in sibship analysis: a pilot study in Indian subjects. *J Forensic Leg Med*, *14*(5), 261-265. https://doi.org/10.1016/j.jcfm.2006.08.002

Hai, Y., Chong, W., Eisenbrey, J. R., & Forsberg, F. (2022). Network Meta-Analysis: Noninvasive Imaging Modalities for Identifying Clinically Significant Portal Hypertension. *Digestive Diseases and Sciences*, *67*(7), 3313-3326. https://doi.org/10.1007/s10620-021-07168-y

Jafarzadeh, S. R., Nowrouzian, I., Khaki, Z., Ghamsari, S. M., & Adibhashemi, F. (2004). The sensitivities and specificities of total plasma protein and plasma fibrinogen for the diagnosis of traumatic reticuloperitonitis in cattle. *Prev Vet Med*, *65*(1-2), 1-7. https://doi.org/10.1016/j.prevetmed.2004.07.004

Jain, N. B., Luz, J., Higgins, L. D., Dong, Y., Warner, J. J., Matzkin, E., & Katz, J. N. (2017). The Diagnostic Accuracy of Special Tests for Rotator Cuff Tear: The ROW Cohort Study. *Am J Phys Med Rehabil*, *96*(3), 176-183. https://doi.org/10.1097/phm.0000000000000566

Landsheer, J. A. (2020). Impact of the Prevalence of Cognitive Impairment on the Accuracy of the Montreal Cognitive Assessment: The Advantage of Using two MoCA Thresholds to Identify Error-prone Test Scores. *Alzheimer Dis Assoc Disord*, *34*(3), 248-253. https://doi.org/10.1097/wad.0000000000000365

Lee, K. H., Lim, K. Y., Suh, Y. J., Hur, J., Han, D. H., Kang, M. J., Choo, J. Y., Kim, C., Kim, J. I., Yoon, S. H., Lee, W., & Park, C. M. (2019). Diagnostic Accuracy of Percutaneous Transthoracic Needle Lung Biopsies: A Multicenter Study. *Korean J Radiol*, *20*(8), 1300-1310. https://doi.org/10.3348/kjr.2019.0189

Li, Q., Zhang, L., Liao, X., Tang, S., & Li, Z. (2021). Ultrasound-guided percutaneous needle biopsies of peripheral pulmonary lesions: diagnostic efficacy and risk factors for diagnostic failure. *Annals of Palliative Medicine*, *10*(9), 9772-9783. https://apm.amegroups.com/article/view/79465

Link, T. M., Sell, C. A., Masi, J. N., Phan, C., Newitt, D., Lu, Y., Steinbach, L., & Majumdar, S. (2006). 3.0 vs 1.5T MRI in the detection of focal cartilage pathology – ROC analysis in an experimental model. *Osteoarthritis and Cartilage*, *14*(1), 63-70. https://doi.org/https://doi.org/10.1016/j.joca.2005.08.002

Min, J. J., Kim, G., Kim, E., & Lee, J. H. (2016). The diagnostic validity of clinical airway assessments for predicting difficult laryngoscopy using a grey zone approach. *J Int Med Res*, *44*(4), 893-904. https://doi.org/10.1177/0300060516642647

Nasis, A., Leung, M. C., Antonis, P. R., Cameron, J. D., Lehman, S. J., Hope, S. A., Crossett, M. P., Troupis, J. M., Meredith, I. T., & Seneviratne, S. K. (2010). Diagnostic Accuracy of Noninvasive Coronary Angiography With 320-Detector Row Computed Tomography. *The American Journal of Cardiology*, *106*(10), 1429-1435. https://doi.org/https://doi.org/10.1016/j.amjcard.2010.06.073

Ooi, G. J., Earnest, A., Kemp, W. W., Burton, P. R., Laurie, C., Majeed, A., Johnson, N., McLean, C., Roberts, S. K., & Brown, W. A. (2018). Evaluating feasibility and accuracy of non-invasive tests for nonalcoholic fatty liver disease in severe and morbid obesity. *International Journal of Obesity*, *42*(11), 1900-1911. https://doi.org/10.1038/s41366-018-0007-3

Philbrick, J. T., Shumate, R., Siadaty, M. S., & Becker, D. M. (2007). Air Travel and Venous Thromboembolism: A Systematic Review. *Journal of General Internal Medicine*, *22*(1), 107-114. https://doi.org/10.1007/s11606-006-0016-0

Ropka, M. E., Keim, J., & Philbrick, J. T. (2010). Patient decisions about breast cancer chemoprevention: a systematic review and meta-analysis. *J Clin Oncol*, *28*(18), 3090-3095. https://doi.org/10.1200/jco.2009.27.8077

Ropka, M. E., Wenzel, J., Phillips, E. K., Siadaty, M., & Philbrick, J. T. (2006). Uptake Rates for Breast Cancer Genetic Testing: A Systematic Review. *Cancer Epidemiology, Biomarkers & Prevention*, *15*(5), 840-855. https://doi.org/10.1158/1055-9965.Epi-05-0002

Rosendahl, C., Tschandl, P., Cameron, A., & Kittler, H. (2011). Diagnostic accuracy of dermatoscopy for melanocytic and nonmelanocytic pigmented lesions. *Journal of the American Academy of Dermatology*, *64*(6), 1068-1073. https://doi.org/https://doi.org/10.1016/j.jaad.2010.03.039

Staufer, K., Halilbasic, E., Spindelboeck, W., Eilenberg, M., Prager, G., Stadlbauer, V., Posch, A., Munda, P., Marculescu, R., Obermayer-Pietsch, B., Stift, J., Lackner, C., Trauner, M., & Stauber, R. E. (2019). Evaluation and comparison of six noninvasive tests for prediction of significant or advanced fibrosis in nonalcoholic fatty liver disease. *United european gastroenterology journal*, *7*(8), 1113-1123. https://doi.org/https://doi.org/10.1177/2050640619865133

Stojadinovic, M., Vukovic, I., Ivanovic, M., Stojadinovic, M., Milovanovic, D., Pantic, D., & Jankovic, S. (2020). Optimal threshold of the prostate health index in predicting aggressive prostate cancer using predefined cost–benefit ratios and prevalence. *International Urology and Nephrology*, *52*(5), 893-901. https://doi.org/10.1007/s11255-019-02367-z

Subhas, N., Sakamoto, F. A., Mariscalco, M. W., Polster, J. M., Obuchowski, N. A., & Jones, M. H. (2012). Accuracy of MRI in the Diagnosis of Meniscal Tears in Older Patients. *American Journal of Roentgenology*, *198*(6), W575-W580. https://doi.org/10.2214/AJR.11.7226

Virgili, G., Menchini, F., Dimastrogiovanni, A. F., Rapizzi, E., Menchini, U., Bandello, F., & Chiodini, R. G. (2007). Optical Coherence Tomography versus Stereoscopic Fundus Photography or Biomicroscopy for Diagnosing Diabetic Macular Edema: A Systematic Review. *Investigative ophthalmology & visual science*, *48*(11), 4963-4973. https://doi.org/10.1167/iovs.06-1472

Vu, J. P., Yamin, G., Reyes, Z., Shin, A., Young, A., Litvan, I., Xie, P., & Obrzut, S. (2021). Assessment of Motor Dysfunction with Virtual Reality in Patients Undergoing [(123)I]FP-CIT SPECT/CT Brain Imaging. *Tomography*, *7*(2), 95-106. https://doi.org/10.3390/tomography7020009

Wardziak, Ł., Kruk, M., Pleban, W., Demkow, M., Rużyłło, W., Dzielińska, Z., & Kępka, C. (2019). Coronary CTA enhanced with CTA based FFR analysis provides higher diagnostic value than invasive coronary angiography in patients with intermediate coronary stenosis. *Journal of cardiovascular computed tomography*, *13*(1), 62-67. https://doi.org/https://doi.org/10.1016/j.jcct.2018.10.004
