# Supplementary figures and images for: Missing values and inconclusive results in diagnostic studies – A scoping review of methods
Source: Stat Methods Med Res. 2023 Aug 9;32(9):1842–55. doi: 10.1177/09622802231192954 (PMC10540494; doi:10.1177/09622802231192954)

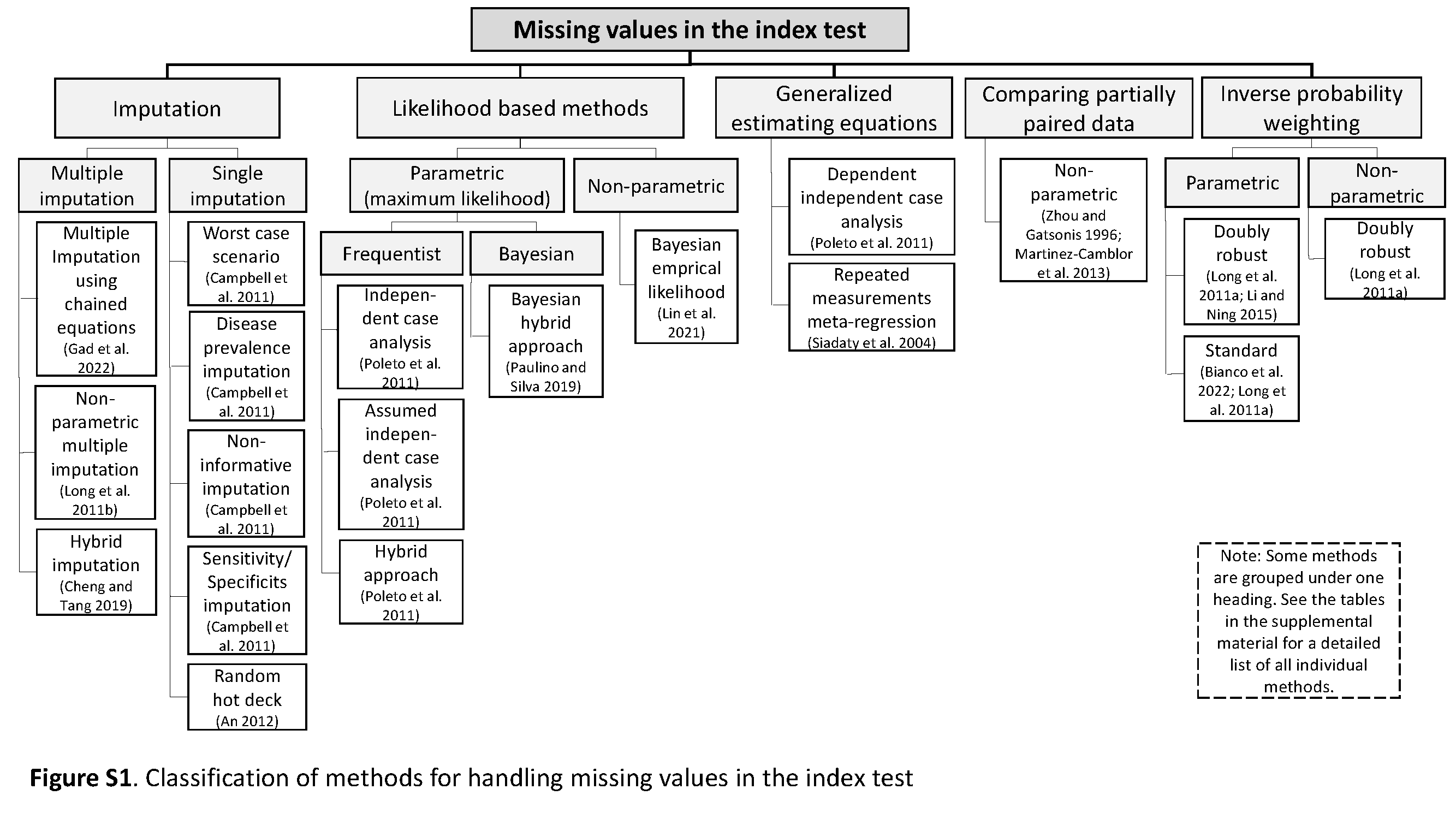

Supplement: sj-tif-3-smm-10.1177_09622802231192954 - Supplemental material for Missing values and inconclusive results in diagnostic studies – A scoping review of methods [file sj-tif-3-smm-10.1177_09622802231192954.tif]

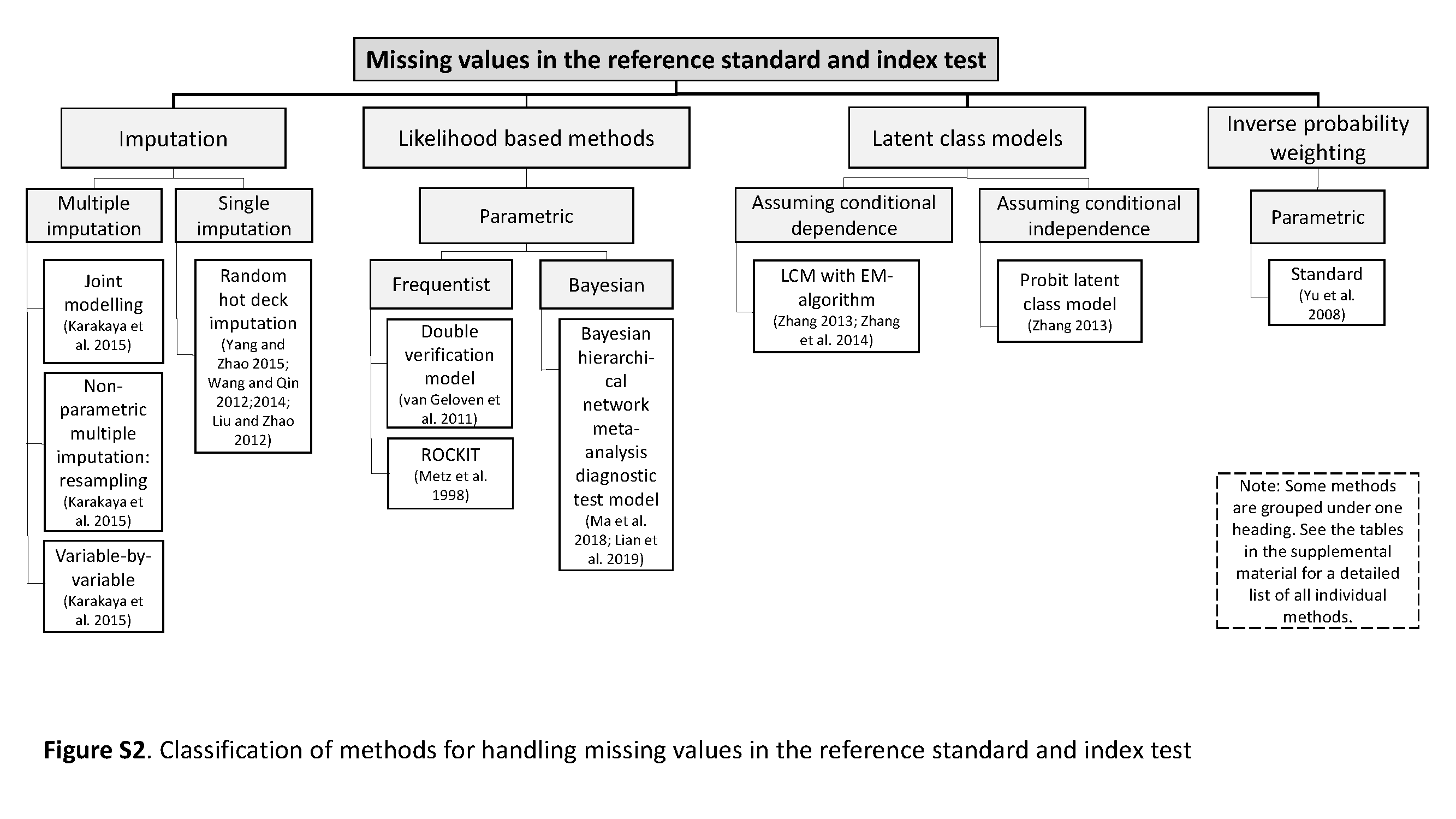

Supplement: sj-tif-4-smm-10.1177_09622802231192954 - Supplemental material for Missing values and inconclusive results in diagnostic studies – A scoping review of methods [file sj-tif-4-smm-10.1177_09622802231192954.tif]

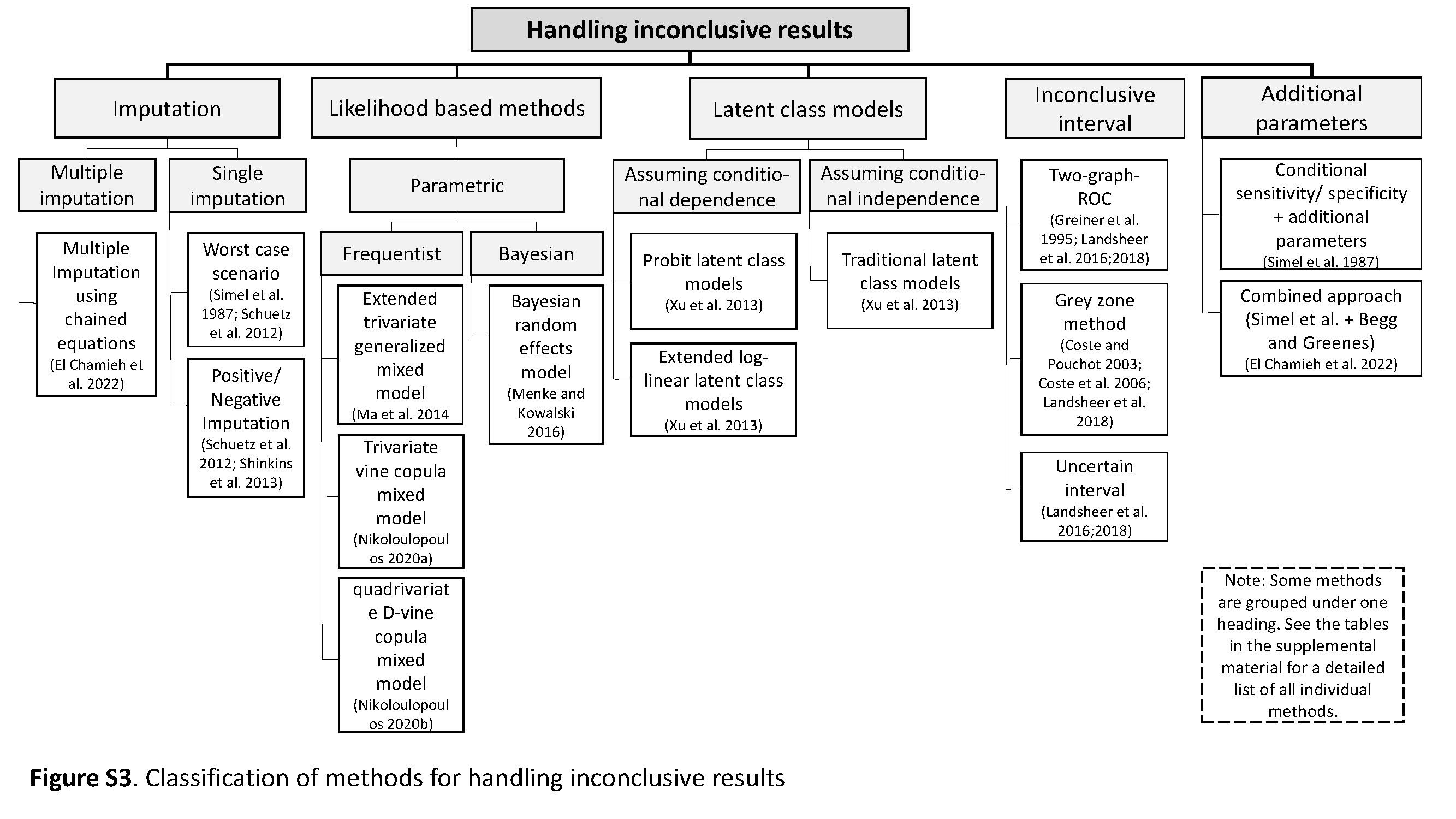

Supplement: sj-tif-5-smm-10.1177_09622802231192954 - Supplemental material for Missing values and inconclusive results in diagnostic studies – A scoping review of methods [file sj-tif-5-smm-10.1177_09622802231192954.tif]
